# suPAR links a dysregulated immune response to tissue inflammation and sepsis-induced acute kidney injury

**DOI:** 10.1172/jci.insight.165740

**Published:** 2023-04-10

**Authors:** Christian Nusshag, Changli Wei, Eunsil Hahm, Salim S. Hayek, Jing Li, Beata Samelko, Christoph Rupp, Roman Szudarek, Claudius Speer, Florian Kälble, Matthias Schaier, Florian Uhle, Felix C.F. Schmitt, Mascha O. Fiedler, Ellen Krautkrämer, Yanxia Cao, Ricardo Rodriguez, Uta Merle, Jesper Eugen-Olsen, Martin Zeier, Markus A. Weigand, Christian Morath, Thorsten Brenner, Jochen Reiser

**Affiliations:** 1 Department of Internal Medicine, RUSH University Medical Center, Chicago, Illinois, USA.; 2 Department of Nephrology, Heidelberg University Hospital, Heidelberg, Germany.; 3 Department of Medicine, Division of Cardiology, University of Michigan, Ann Arbor, Michigan, USA.; 4 Department of Anesthesiology, and; 5 Department of Gastroenterology, Heidelberg University Hospital, Heidelberg, Germany.; 6 Department of Clinical Research, Copenhagen University Hospital Hvidovre, Hvidovre, Denmark.; 7 Department of Anesthesiology and Intensive Care Medicine, University Hospital Essen, University Duisburg-Essen, Essen, Germany.

**Keywords:** Inflammation, Nephrology, Clinical practice, Diagnostics, T cells

## Abstract

Acute kidney injury (AKI) secondary to sepsis results in poor outcomes and conventional kidney function indicators lack diagnostic value. Soluble urokinase plasminogen activator receptor (suPAR) is an innate immune–derived molecule implicated in inflammatory organ damage. We characterized the diagnostic ability of longitudinal serum suPAR levels to discriminate severity and course of sepsis-induced AKI (SI-AKI) in 200 critically ill patients meeting Sepsis-3 criteria. The pathophysiologic relevance of varying suPAR levels in SI-AKI was explored in a polymicrobial sepsis model in WT, (s)uPAR-knockout, and transgenic suPAR-overexpressing mice. At all time points studied, suPAR provided a robust classification of SI-AKI disease severity, with improved prediction of renal replacement therapy (RRT) and mortality compared with established kidney biomarkers. Patients with suPAR levels of greater than 12.7 ng/mL were at highest risk for RRT or death, with an adjusted odds ratio of 7.48 (95% CI, 3.00–18.63). suPAR deficiency protected mice against SI-AKI. suPAR-overexpressing mice exhibited greater kidney damage and poorer survival through inflamed kidneys, accompanied by local upregulation of potent chemoattractants and pronounced kidney T cell infiltration. Hence, suPAR allows for an innate immune–derived and kidney function–independent staging of SI-AKI and offers improved longitudinal risk stratification. suPAR promotes T cell–based kidney inflammation, while suPAR deficiency improves SI-AKI.

## Introduction

Acute kidney injury (AKI) is a serious complication in critically ill patients, and sepsis is the leading cause, accounting for 30%–50% of cases (1–3). At the time of sepsis diagnosis, however, the clinical course and prognosis of sepsis-induced AKI (SI-AKI) is unclear. The current standard for staging AKI — using the functional parameters serum creatinine (SCr) and urine output — only allow for retrospective clarification of true AKI severity (2, 4). Furthermore, neither SCr nor urine output correlate directly with underlying pathophysiological drivers or extent of kidney tissue damage, limiting their value for assessing further disease progression and clinical decision-making, such as initiation of renal replacement therapy (RRT) (5–8). This is particularly true for sepsis, where the disease activity evolves/differs over time and contrasts with AKI after major surgery, where a single, distinct kidney insult can be identified.

In this context, a newly proposed AKI definition recently suggested the combination of functional biomarkers with biomarkers of kidney damage (9). The latter are thought to be released in response to kidney stress or injury into blood or urine. Their additive value relies on the concept that the injury biomarker concentration more accurately reflects the extent of kidney damage than functional biomarkers. Traditional biomarkers such as urinary albumin, but also novel markers such as the urinary product of the 2 tubular stress and cell cycle arrest biomarker tissue inhibitor of metalloproteinases 2 and insulin-like growth factor binding protein 7 (TIMP2•IGFBP7), or serum levels of neutrophil gelatinase–associated lipocalin (NGAL) or kidney injury molecule 1 (KIM-1) belong in this category (4, 9). In early sepsis, however, severity of organ dysfunction is not uniquely linked to substantial cell death (10, 11). In fact, the severity of ongoing inflammatory stimuli related to a dysregulated host immune response to infection represents a key component for early organ dysfunction in sepsis (10). Pathophysiological drivers of the underlying molecular processes may therefore qualify as robust prognostic and diagnostic biomarkers as well as therapeutic targets for septic organ dysfunction.

We and others have recently identified soluble urokinase plasminogen activator receptor (suPAR) as an indicator for systemic inflammatory conditions and immune mediator of inflammatory organ damage in a diverse range of clinical contexts (12–18). uPAR is a glycosylphosphatidylinositol-anchored protein that is upregulated following bacterial infection and is expressed on a variety of cells, mainly innate immune cells such as monocytes and neutrophils (19–21). The activation of the uPAR system is linked to inflammation, innate immune cell activation, and cell migration (15, 20–22). Inflammatory stimuli generate suPAR by cleavage of its membrane-bound form, uPAR (20, 21). Especially in critically ill patients with inflammatory diseases, high blood suPAR levels are closely linked to acute organ dysfunction and AKI in particular (12–14, 23, 24). This suggests a fundamental role for suPAR as a pathophysiological interface between systemic inflammatory states and resulting organ damage. The specific physiologic role of suPAR is, however, unclear.

We hypothesized that suPAR — functioning as a direct pathophysiological driver of kidney tissue inflammation in sepsis — could serve as a reliable diagnostic and prognostic biomarker for SI-AKI. To test our hypothesis, we examined the diagnostic value and longitudinal kinetics of blood suPAR levels in 200 critically ill patients with sepsis and compared them to functional and widely established damage biomarkers. To further elucidate the pathophysiological consequences of high blood suPAR levels on kidney function, tissue damage, and kidney tissue inflammation, we established an experimental polymicrobial sepsis model, comparing C57BL/6 WT, uPAR-knockout (uPAR-KO, deficient in suPAR), and a transgenic mouse strain artificially overexpressing full-length suPAR in blood (msuPAR1-OE).

## Results

### Patient characteristics and outcomes.

**Figure 1:**
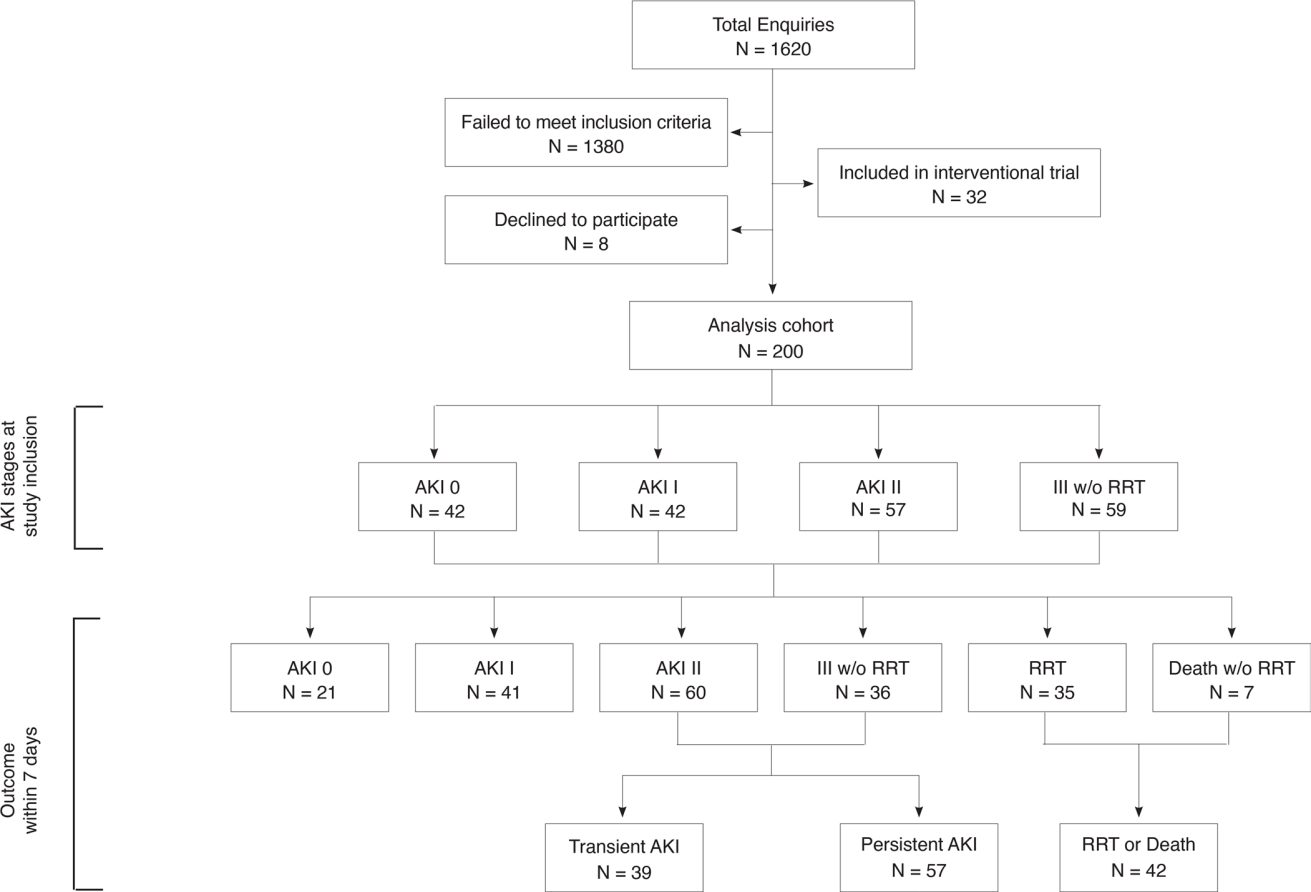
Flow chart of study design and data analysis. AKI, acute kidney injury according to KDIGO criteria; RRT, renal replacement therapy.

Of 1,620 patients assessed for eligibility, 200 patients met the inclusion criteria and were enrolled in the study within an average of 9.6 hours after ICU admission. At enrollment, 79% of patients had SI-AKI (Figure 1). When looking at maximum AKI stages within 7 days of sepsis diagnosis, 21 patients had no AKI, while 41, 60, and 36 patients developed maximum AKI stages of mild, moderate, and severe AKI, respectively, without requiring RRT. Forty-two patients met the primary outcome of RRT or death. Of the 96 patients who experienced moderate or severe AKI without need for RRT, 39 had transient AKI and 57 had persistent AKI (Figure 1).

Comparing patients who died or required RRT with the remaining cohort, these patients were more likely to have higher disease severity, greater fluid overload, septic shock, worse kidney function parameters, and higher serum suPAR, NGAL, and urinary TIMP2•IGFBP7 levels at study inclusion (Table 1). Baseline demographics, preexisting comorbidities, baseline creatinine prior sepsis, and inflammatory parameters did not differ between the 2 groups. Overall, 19 patients (9.5%) died within 7 days of enrollment (12 patients on RRT, 7 without RRT). The 30-day all-cause mortality was 22.5% (45 patients), with higher mortality in patients who required RRT (51.4%) than in patients who did not (16.6%). Patient characteristics and outcomes stratified by suPAR quartiles are shown in Supplemental Table 1; supplemental material available online with this article; https://doi.org/10.1172/jci.insight.165740DS1

### Kinetics of human suPAR in sepsis and its diagnostic value for outcome prediction.

**Figure 2:**
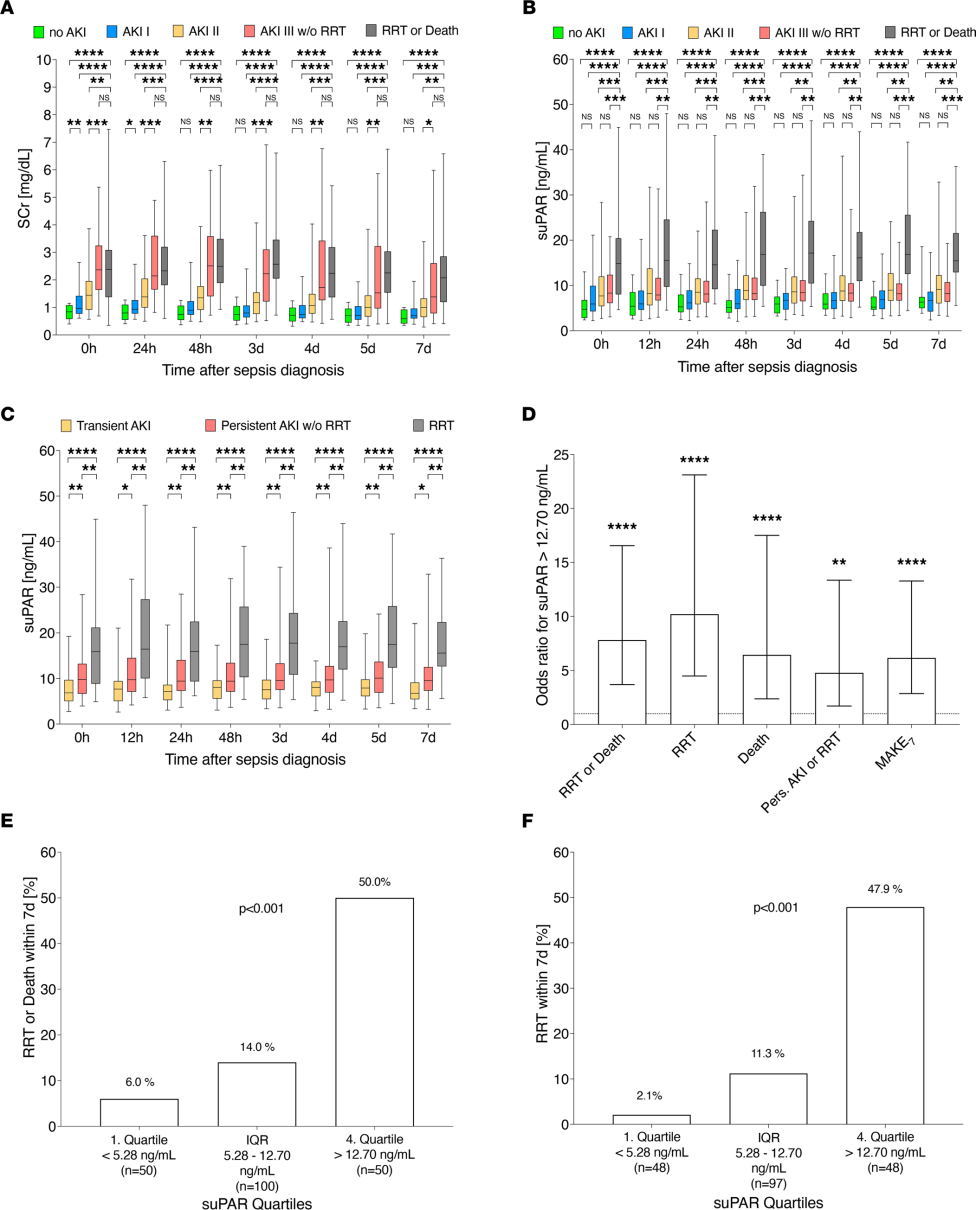
Blood suPAR levels discriminate between maximum AKI stages, varying AKI courses, and poor (kidney) outcome in human sepsis at any time within 7 days of sepsis diagnosis. (**A**–**C**) Outcome-related course of serum creatinine (SCr) and soluble urokinase plasminogen activator receptor (suPAR) over 7 days after sepsis diagnosis (0 hours, *n* = 200; 12 hours, *n* = 190; 24 hours, *n* = 190; 48 hours, *n* = 186; 3 days, *n* = 177; 4 days, *n* = 175; 5 days, *n* = 167; 7 days, *n* = 155) and (**D**–**F**) outcome in relation to suPAR quartiles at baseline. *P ≤ 0.05, **P ≤ 0.01, ***P ≤ 0.001, ****P ≤ 0.0001. NS, *P* > 0.05. AKI, acute kidney injury; IQR, interquartile range; MAKE_7_, major adverse kidney events within 7 days of sepsis diagnosis; RRT, renal replacement therapy. Data are reported as box-and-whisker plots (interquartile range, minimum to maximum) (**A**–**C**), unadjusted odds ratio (95% CI) (**D**), and percentage (**E** and **F**). One-way ANOVA (**A**–**C**) and χ^2^ (**E** and **F**) tests were used for group comparisons.

SCr values did not allow diagnostic differentiation of patients who required RRT or died from patients with severe AKI not requiring RRT (Figure 2A). In contrast, suPAR levels were significantly higher in patients who required RRT or died than those in other AKI stages (Figure 2B) at any time point within 7 days of sepsis diagnosis. suPAR further distinguished between the critical AKI subsets, none-to-mild AKI versus moderate-to-severe AKI not requiring RRT at each time point studied (Figure 2B). Examining the disease course of AKI, suPAR levels were able to indicate the divergent AKI courses transient AKI, persistent AKI, and AKI requiring RRT at any time point within 7 days after enrollment (Figure 2C). When further evaluating suPAR levels at baseline, the higher the suPAR levels, the more likely patients were to require RRT or to die (Figure 2, E and F). Patients in the highest suPAR quartile (suPAR > 12.70 ng/mL) were at highest risk for poor outcomes (Figure 2D). The increased odds for RRT or death and major adverse kidney events within 7 days of sepsis diagnosis (MAKE_7_) were preserved after adjustment for confounders such SCr levels at enrollment or disease severity, represented by SOFA score at enrollment (Table 2). Even when patients with moderate or severe AKI at the time of enrollment were analyzed exclusively, suPAR remained a superior risk classifier for studied outcomes (Supplemental Table 2).

Accordingly, for the prediction of RRT or death, MAKE_7_, and RRT, suPAR alone showed the highest AUCs compared with the widely established kidney biomarkers SCr, albuminuria, and proteinuria, and even compared with the newer kidney damage biomarkers NGAL, TIMP2•IGFBP7, and KIM-1 alone (Table 3 and Supplemental Table 3). The combination of SCr and suPAR further improved outcome prediction performance, resulting in the highest AUC of all combinations of kidney injury biomarkers with SCr. SCr together with suPAR even statistically outperformed the biomarker combinations “SCr + Albuminuria” and “SCr + KIM-1,” and was more or less on par with SCr combined with NGAL (Table 3 and Supplemental Table 3). In addition, with an AUC of 0.78 (95% CI 0.67–0.89), suPAR showed superior performance in predicting 7-day mortality compared with other kidney biomarkers (Supplemental Table 4). Finally, in a model that includes baseline SCr, NGAL, KIM-1, TIMP2•IGFBP7, and albuminuria to predict RRT or death, the AUC was 0.83 (95% CI 0.76–0.90) and further increased to 0.85 (95% CI 0.79–0.92) by adding suPAR to the model.

### Murine sepsis and AKI.

**Figure 3:**
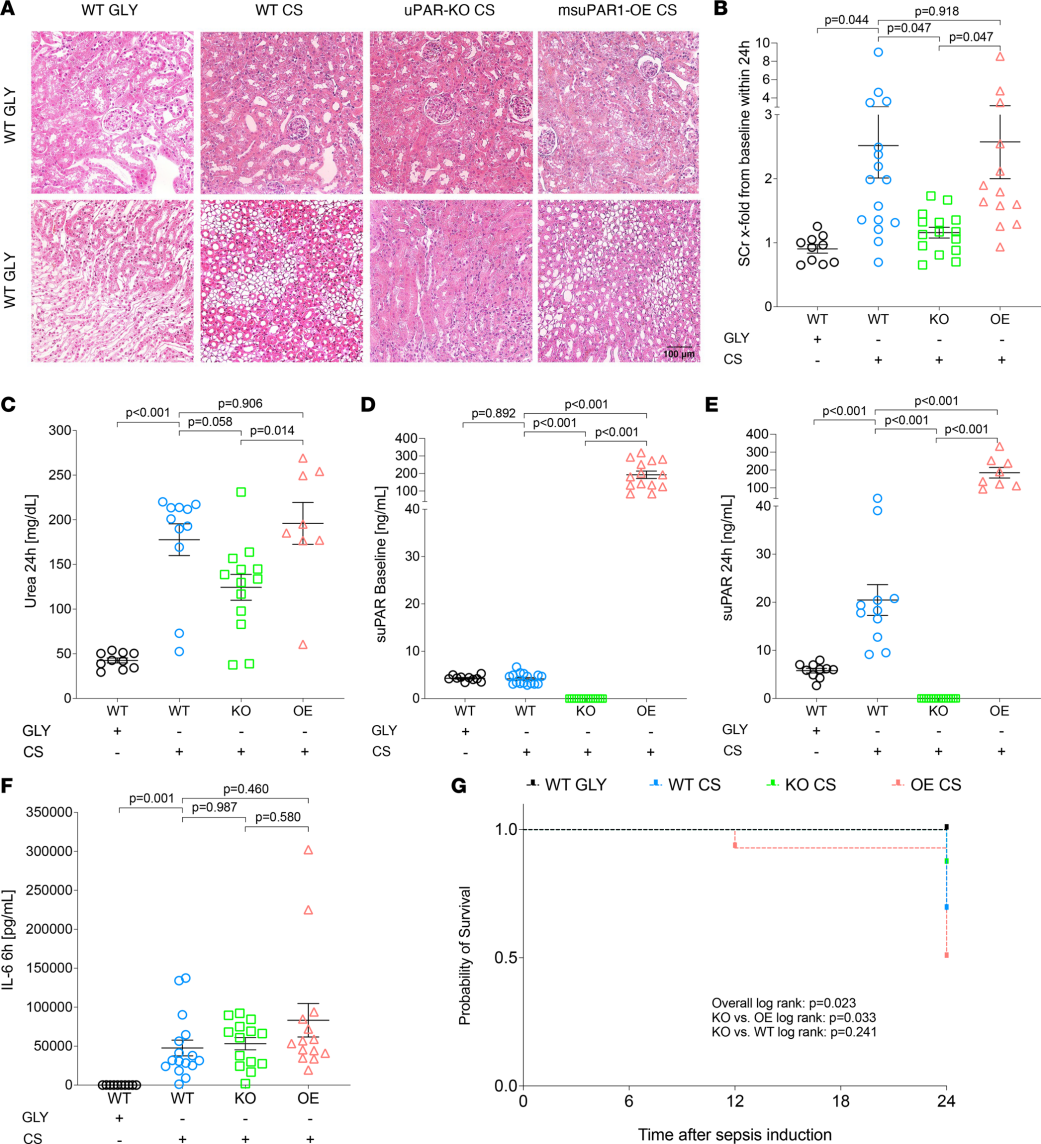
Elevated blood levels of suPAR are associated with enhanced kidney tissue damage, kidney function impairment, and poor (kidney) outcome in murine sepsis. Sepsis was induced via i.p. injection of 250 μL cecal slurry (CS) in C57BL/6 WT (*n* = 16), uPAR-knockout (KO, *n* = 15), and transgenic C57BL/6 with overexpression of suPAR (OE, *n* = 14). Glycerol (GLY, 15%) served as control in WT (vehicle control, *n* = 10). (**A**) H&E staining of kidneys from different mouse strains 24 hours after sepsis induction. Original magnification, ×40. Scale bar: 100 μm. (**B**) Maximum serum creatinine (SCr) changes from baseline within 24 hours and (**C**) urea 24 hours after sepsis induction. (**D**) suPAR levels at baseline and (**E**) 24 hours after sepsis induction. (**F**) IL-6 levels 6 hours after sepsis induction. (**G**) Survival analysis of different mouse strains. Survival: WT GLY, 10/10 (100%); WT CS, 11/16 (69%); KO CS, 13/15 (87%); OE CS, 7/14 (50%). Data are reported as mean ± SEM. One-way ANOVA test was used for group comparisons (**B**–**F**), and the Kaplan-Meier method and log-rank testing were used for survival analyses (**G**).

In critically ill patients with sepsis, suPAR best predicted death or AKI requiring RRT. The question arises whether suPAR is merely a systemic inflammatory biomarker or whether high suPAR levels are directly responsible for poor outcomes as pathogenic driver of SI-AKI. To further address this question, we examined the effects of blood suPAR levels on kidney function and tissue damage/inflammation in a polymicrobial sepsis model, in WT, uPAR-KO, and msuPAR1-OE mice with physiological, depleted, or genetically elevated (independent of inflammatory conditions) blood suPAR levels, respectively. Before sepsis induction, SCr and urea levels were comparable between different mouse strains (Supplemental Figure 1). When inducing polymicrobial sepsis by injecting 250 μL of cecal slurry (CS) intraperitoneally, WT and msuPAR1-OE mice experienced more severe AKI compared with uPAR-KO mice deficient in suPAR (Figure 3, A–C, and Supplemental Figure 1) within 24 hours of sepsis induction. This was evidenced by more pronounced kidney tissue damage, and higher SCr and urea levels in septic WT and msuPAR1-OE compared with uPAR-KO. Mice receiving 15% glycerol (WT GLY) as vehicle control did not develop AKI (Figure 3 and Supplemental Figure 1). As shown by H&E staining, kidney structure was largely unaltered in WT GLY mice, except for mild interstitial edema in the medulla. In contrast, loss of brush border could be seen in some proximal tubules in the cortex of WT CS mice with foci of acute tubular injury in the outer strips of medullary tissue, as indicated by mild epithelial simplification and cell detachment. Compared with WT CS, the morphological changes in msuPAR1-OE mice receiving CS were similar but with more pronounced tubular vacuolization in the cortex and more severe tubular injury in the outer medullary tissues. uPAR-KO mice were largely protected from CS effects, with no major morphological changes in the medulla and only mild tubular changes in the cortex (Figure 3A). The number of apoptotic cells in kidney tissue was not strongly pronounced but significantly higher in msuPAR1-OE CS compared with uPAR-KO CS and WT CS, while no apparent apoptosis was seen in WT GLY mice (Supplemental Figure 2). At baseline, mean blood suPAR levels (±SD) in WT, uPAR-KO, and msuPAR1-OE strains were 4.29 ng/mL (±0.58), 0 ng/mL (±0.00), and 192.60 ng/mL (±79.44), respectively (Figure 3D). After sepsis induction, suPAR levels significantly increased in WT CS mice, whereas no relevant changes were seen in WT GLY, uPAR-KO CS, and msuPAR1-OE CS mice compared to baseline (Figure 3E and Supplemental Figure 3). Though more pronounced functional and ultrastructural kidney impairment was observed in strains with elevated blood suPAR levels (WT CS, msuPAR1-OE CS), the extent of systemic inflammation, as indicated by IL-6 levels, was comparable between mouse strains investigated (Figure 3F and Supplemental Figure 3). Finally, uPAR-KO was associated with improved survival compared with msuPAR1-OE mice after 24 hours of sepsis induction (Figure 3G).

### Characterization of kidney tissue inflammation in experimental SI-AKI and untreated mouse strains.

**Figure 4:**
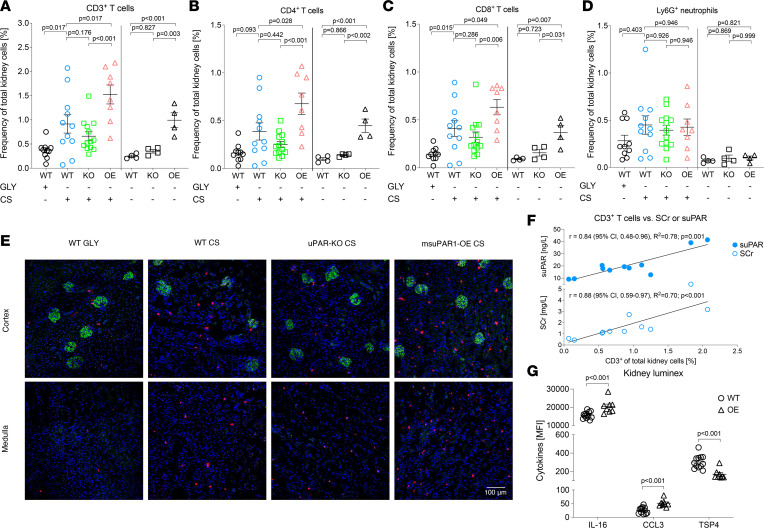
Characterization of kidney leukocyte subsets in C57BL/6 WT, uPAR-KO, and transgenic C57BL/6 with overexpression of suPAR reveals a link between increased blood suPAR levels and kidney T cell accumulation, kidney function impairment, and local upregulation of inflammatory cytokines. (**A**–**D**) Strain-dependent characterization of leukocyte subsets by flow cytometry after 24 hours of sepsis induction (left) via i.p. injection of 250 μL cecal slurry (CS) and untreated mice (right). Injection of 15% glycerol (GLY) served as control (vehicle solution). (**E**) Exemplary double immunofluorescent staining for podocin (green) and CD8^+^ T cells (red) of kidney tissue from different mouse strains after 24 hours of sepsis. Nuclei were stained with DAPI (blue). Spleen tissue served as positive (primary and secondary antibody) and negative (secondary antibody only) control (data not shown). To quantify kidney immune cell aggregation, the mean cell number was determined from 10 representative high-power fields per animal (see supplemental material). Original magnification, ×40. Scale bar: 100 μm. (**F**) Correlation analysis of kidney T cells and corresponding blood serum creatinine (SCr) and suPAR levels in WT sepsis. (**G**) Kidney Luminex analysis of homogenized kidney tissue of untreated WT and suPAR-OE mice. CCL, C-C motif chemokine ligand 3; MFI, median fluorescence intensity; TSP4, thrombospondin 4. Data are reported as mean ± SEM. One-way ANOVA test was used for multiple group comparisons (**A**–**D**), correlations were assessed by using Pearson’s correlation analysis (**F**), and 2-tailed Student’s *t* test was used for pairwise comparisons (**G**).

Since the uPAR system is known to play a critical role in cell migration and inflammation, we further investigated the characteristics and extent of suPAR-related kidney tissue inflammation in experimental sepsis. To evaluate the effect of varying blood suPAR levels in a noninflammatory environment, untreated mouse strains were analyzed in the same manner. After 24 hours of sepsis, flow cytometry of digested kidneys showed a significantly higher frequency of CD45^+^ leukocytes in the kidneys of msuPAR1-OE CS compared with WT CS and uPAR-KO CS mice (Supplemental Figure 4). This significant difference was largely driven by CD3^+^ T cells (Figure 4, A–C, and Supplemental Figure 4), which were significantly higher in msuPAR1-OE CS compared with WT CS and uPAR-KO CS mice, and higher in WT CS compared with WT GLY mice. The same observation was appreciated for CD4^+^ and CD8^+^ T cells. In contrast, no strain- or suPAR-dependent differences in cell frequencies for Ly6G^+^ neutrophils, CD11c^+^ dendritic cells, NK1.1^+^ cells, and CD3^+^NK1.1^+^ T cells were observed in CS-treated mice (Figure 4D and Supplemental Figure 4). Surprisingly, treatment of WT mice with glycerol alone resulted in an increase in Ly6G^+^ and NK1.1^+^ kidney cells, comparable to CS-treated mice but without impairment of kidney function (Figure 3, B and C, Figure 4D, and Supplemental Figure 4). The only other significant finding was a higher frequency of Ly6C^hi^ monocytes in msuPAR1-OE CS mice compared with uPAR-KO CS mice, without reaching statistical significance compared with WT CS (Supplemental Figure 4). Furthermore, increased numbers of especially kidney T cells, but also monocytes correlated strongly with the extent of systemic suPAR and SCr elevation in WT CS mice, whereas a moderate or no correlation with SCr or suPAR levels was observed for NK1.1^+^ cells and Ly6G^+^ neutrophils, respectively (Figure 4F and Supplemental Figure 5). Even more remarkably, in untreated msuPAR1-OE mice, the frequency of CD3^+^, CD4^+^, CD8^+^, and NK1.1^+^ kidney cells equaled those previously observed in WT CS mice despite the absence of a systemic inflammatory stimulus (Figure 4, A–C, and Supplemental Figure 4). This was accompanied by a significant increase in local IL-16 and C-C motif chemokine ligand 3 (CCL3) levels, and decreased levels of thrombospondin 4 (TSP4) in kidney tissues of untreated msuPAR1-OE compared with untreated WT (Figure 4G and Supplemental Figure 6). Kidney function, however, was not acutely affected by the mere presence of these cells and altered cytokines in untreated msuPAR1-OE mice.

The characteristic suPAR-dependent kidney immune cell accumulation was additionally confirmed by immunofluorescent staining in septic and untreated mice for Ly6G/C^+^, CD4^+^, and CD8^+^ kidney cells (Figure 4E and Supplemental Figures 7–9). Both CD4^+^ and CD8^+^ cells were found in the kidney cortex and medulla, with CD8^+^ cells being slightly more abundant and localized in the corticomedullary junction than CD4^+^ cells in CS-treated and untreated mice.

## Discussion

AKI is a common finding in critically ill patients at the time of sepsis diagnosis, especially when the Sepsis-3 criteria are applied (10, 25, 26). Unfortunately, early and robust characterization of the extent and disease course of kidney injury remains an issue in clinical practice. Thus, new, innovative biomarkers with improved prognostic and diagnostic abilities and, in the best scenario, with therapeutic implications will have immense clinical impact (9, 27).

In the current study, we report that circulating levels of the immune-derived glycoprotein suPAR can discriminate between clinically relevant subsets of SI-AKI severity (highest AKI stage) and patients that died or required RRT at any time point within 7 days of sepsis diagnosis. In addition, suPAR was not only a longitudinal indicator of maximal disease severity over 7 days, but also distinguished between different SI-AKI courses such as transient AKI, persistent AKI, and AKI requiring RRT at any time point studied. Thereby, suPAR represents a unique biomarker to combine these diagnostic capabilities, holding out the prospect for meaningful biomarker-guided AKI management in sepsis (9). These special characteristics and kinetics clearly separate suPAR from traditional kidney function biomarkers as well as recently developed damage biomarkers whose performance — mainly based on their short half-life — is largely dependent on the timing of measurement (9, 14, 28, 29). Further highlighting the clinical relevance of suPAR, baseline suPAR values showed the highest AUC for predicting all studied endpoints, compared with traditional and all newer available kidney biomarkers. In combination with SCr, outcome prediction increased further, reaching the highest AUC of all biomarker combinations studied. The combination of suPAR and SCr even statistically outperformed combinations of SCr with damage biomarkers such as albuminuria or KIM-1, and was more or less equivalent to SCr combined with NGAL, the latter, however, being an additional leukocyte- and liver-derived biomarker in sepsis rather than an independent kidney damage biomarker (30–32). Moreover, a model to predict RRT or death that included all kidney biomarkers studied, improved further by adding suPAR to the model, highlighting once more suPAR’s additive diagnostic value.

Our data not only support the relevance of suPAR as a new diagnostic biomarker, but more importantly strengthen the concept that suPAR — in contrast to the above-mentioned biomarkers — is a direct pathophysiological driver involved in SI-AKI. We found pronounced ultrastructural kidney damage, impaired kidney function, and poor survival associated with high blood suPAR levels in a polymicrobial murine sepsis model. While transgenic overexpression of blood suPAR resulted in even more pronounced kidney damage, uPAR-KO (no circulating suPAR) showed strong protective effects, including improved kidney function and survival, clearly linking increased suPAR expression to increased injury in SI-AKI. In line with our data, an experimental study by Kiyan et al. recently demonstrated improved organ function in uPAR-KO mice in sepsis (22). The authors attributed this to decreased Toll-like receptor 4 (TLR4) signaling in uPAR-KO mice via mitigated innate immune response and cytokine release. Though we were able to reproduce the protective effect of uPAR-KO in our sepsis model, systemic levels of cytokines such as IL-6 were not significantly different between the studied strains.

Infiltration of immune cells is considered another pathophysiologic cornerstone of kidney injury (1). While massive neutrophil infiltration is a hallmark of ischemia reperfusion injury–induced (IRI-induced) AKI, data on histopathologic changes in SI-AKI are inconsistent and poorly understood (33–37). We now find that overall kidney immune cell composition in early experimental sepsis (first 24 hours) is characterized by dominant infiltration of CD4^+^ and CD8^+^ T cells, and NK1.1^+^ cells. Particularly, the amount of kidney T cells is closely linked to kidney function impairment and the extent of corresponding systemic suPAR elevation in sepsis. Numbers of kidney NK1.1^+^ cells, however, were less dependent on blood suPAR levels in sepsis. On the other hand, we made the astonishing observation that solely high blood suPAR levels in untreated msuPAR1-OE mice led to upregulated kidney tissue levels of IL-16 and CCL3, with increased frequencies of kidney T cells and NK1.1^+^ previously observed in septic WT mice. Since IL-16 and CCL3 are both potent chemoattractants for T and NK cells (38–40) and known molecules involved in cell-mediated kidney damage, this indicates a distinct role of the suPAR molecule for kidney tissue inflammation and subsequent organ damage (41, 42). Also, urinary IL-16 levels have recently been identified as an indicator of kidney tissue inflammation in lupus nephritis (43). The significance of downregulated TSP4 levels in untreated msuPAR1-OE, however, remains unclear and requires further clarification.

Taken together, our data show that suPAR represents a mediator of kidney tissue inflammation in SI-AKI that triggers and aggravates AKI in the presence of additional inflammatory stimuli. Our finding that untreated msuPAR1-OE mice exhibit inflamed kidney tissue and are known to develop impaired kidney function over time reinforces suPAR’s role as predisposing risk factor for chronic kidney disease (CKD) and AKI of other causes, and is in line with the observation in humans that high suPAR levels predict future decline in glomerular filtration rate (44–46).

In particular, the fact that elevated suPAR levels are capable of fueling kidney tissue inflammation in septic and nonseptic conditions — and that uPAR-KO protects against SI-AKI — identifies suPAR as a key signaling molecule and target for novel kidney-protecting therapies through targeting the immune system.

Further, in contrast to recently developed damage biomarkers such as TIMP2•IGFBP7, suPAR’s direct pathophysiological involvement and long-lasting kinetics provide robust, longitudinal information on the activity of kidney tissue inflammation, kidney damage, and prognosis in SI-AKI. Thus, suPAR differs conceptually from classical biomarkers, showing that an immune-derived molecule can be used for severity grading and prognostic assessment of SI-AKI. Though further research is needed, the cross-linking of suPAR with tissue inflammation may be generalizable to other organs, explaining for the first time to our knowledge why high blood suPAR levels are predictive for inflammatory organ dysfunction of various kinds (12–14, 23, 24).

Lastly, our results suggest a specific role of T cell–mediated kidney damage within the first 24 hours of sepsis. In contrast to neutrophils, differences in kidney T cell numbers correlated strongly with impaired kidney function and ultrastructural kidney damage. In line with this, growing evidence emphasizes the role of T lymphocytes for AKI pathogenesis and magnitude (33, 43, 44). Naive CD4^+^ cells can differentiate into Tregs, and into Th1, Th2, and Th17 cells, producing relevant amounts of IFN-γ, IL-4, and IL-17, respectively. In particular, Th1-dominant immune response patterns seem to result in greater, IFN-γ–driven kidney injury, as highlighted in a study by Rabb et al. (47). In contrast, favoring Th2 signaling pathways with IL-4 production has been reported to be protective against IRI-AKI (48). But, CD8^+^ cells also possess the capacity to harm cellular kidney structures by differentiating into cytotoxic effector cells, which produce relevant amounts of IFN-γ and TNF-α (49–51). In addition, a critical role of Tregs in early sepsis was recently highlighted by a study, in which their depletion had a protective effect in SI-AKI (34). Accordingly, several other studies linked T cells to AKI emergence by showing that depletion of CD4^+^ and CD8^+^ cells protects against cisplatin- and IRI-induced AKI (52–54). However, the kinetics of conventional T cell activation (at least 3 days) is a source of major controversy since it is not congruent with the rapid impairment of kidney function that occurs within the first 24 hours of sepsis (55). This is why faster, antigen-independent mechanisms for T cell activation, as are known for NK1.1^+^ T cells, are coming into focus (56–58). NK1.1^+^ T cells possess the ability to produce large amounts of IFN-γ and IL-17 within hours of activation (56–58). NK1.1^+^ T cells can be activated directly by microbial glycolipids presented on CD1d or indirectly through IL-12 produced by TLR-stimulated dendritic cells (59). There is also evidence that activation of NK1.1^+^ T cells leads to severe damage to kidney endothelial cells and thus significantly impairs kidney function and integrity (60). In our study, however, the frequency of CD3^+^NK1.1^+^ kidney cells was hardly related to the magnitude of kidney injury and suPAR elevation. More recently, Th17 cells have emerged as potential players in the pathophysiology of AKI, but there are still many questions that need to be clarified to assess their true relevance (55, 61). It is also worth noting that most of the results discussed above are derived from IRI-AKI models and may have limited applicability to SI-AKI given its very different pathophysiology (1).

Finally, the sepsis model used may itself have an impact on results, as it has already been shown that artificial endotoxin–based sepsis models may lead to different histopathologic findings than models using live bacteria for sepsis induction (33). The strength of our CS sepsis model is that it induces polymicrobial sepsis that closely resembles human sepsis and, unlike cecal ligation– and puncture-induced sepsis models, allows for reproducible sepsis severity (62).

We recognize the limitations of the single-center design and that presented clinical cutoff values for suPAR need further validation in larger, multicenter cohorts. Nevertheless, its unique longitudinal biomarker properties, and the combination of additional diagnostic benefits over conventional kidney parameters with the experimental proof that suPAR is directly involved in kidney pathologies in sepsis, stands out from most recently developed damage biomarkers. We also acknowledge that the incidence of septic shock and AKI is higher in our cohort, as expected from previous literature and similar populations. This may be based on a selection bias, as the most severely ill patients from our center and adjacent catchment area were admitted to the 2 ICUs where the study was conducted. This fact is underlined by an overall higher disease severity (SOFA score) and a higher incidence of septic shock in our cohort, compared with equivalent studies in septic patients (7). Importantly, the high incidence of SI-AKI does not affect the quality of our analyses, since our outcome measures were clearly predefined. In addition, we have made great efforts to obtain the most precise information on baseline creatinine levels prior sepsis to allow for most accurate AKI staging.

To conclude, the integration of immune-derived molecules such as suPAR for disease grading of SI-AKI represents, to our knowledge, a novel approach and new biomarker category for managing AKI and T cell–driven kidney inflammation. Our findings may have large clinical implication since suPAR testing is widely available and holds great potential for future kidney-protective therapeutics in sepsis.

## Methods

### Study design, definitions, and clinical endpoints.

This prospective, observational study was conducted at Heidelberg University Hospital, Germany. Between May 2017 and September 2019, 200 critically ill patients with positive Sepsis-3 criteria at ICU admission were enrolled and treated according to the newest sepsis guidelines (63). Serum suPAR concentration was measured at 0 hours, 12 hours, 24 hours, 48 hours, 72 hours (3 days), 96 hours (4 days), 120 hours (5 days), and 168 hours (7 days) after the time of sepsis diagnosis. suPAR ELISAs were provided by ViroGates. Serum concentrations of NGAL and KIM-1 and urine concentrations of TIMP2•IGFBP7 were measured at 0 hours (patient enrollment). The primary outcome measure was RRT or death within 7 days starting from the time of sepsis diagnosis. Restrictive RRT criteria were predefined. AKI definition was based on the KDIGO criteria (urine output and SCr criteria) (4). Additional outcomes were RRT, 7-day mortality, transient and persistent AKI, major adverse kidney events within 7 days of sepsis diagnosis (MAKE_7_: combinatory endpoint of RRT, death or persistent AKI), and 30-days all-cause mortality. For more information on secondary outcomes, laboratory methods, detailed inclusion, exclusion and RRT criteria, and definitions of baseline SCr as well as transient and persistent AKI, see the supplemental material.

### Murine sepsis model, tissue analyses, and flow cytometry.

A CS polymicrobial sepsis model was used, as recently described (62). Briefly, using the filtered cecal contents of C57BL/6 mice diluted with sterile water, PBS, and glycerol, a CS stock solution containing 6.31 × 10^4^ colony-forming units (CFU) per mL in 15% glycerol was prepared and stored in cryovials at –80°C. For sepsis induction, CS cryovials were thawed and immediately used for intraperitoneal injection of different mouse strains (9–12 weeks old, male). For each experiment, constant bacterial viability was assured for comparable sepsis severity. The optimal CS dose of 250 μL was determined by a titration study that demonstrated a strong correlation between blood suPAR levels, progressive kidney function impairment, poor survival, and the number of positive blood cultures with increasing CS doses in C57BL/6 WT (Supplemental Figure 10). Glycerol (15%) was used as a control to exclude potential glycerol-related effects regarding kidney function and damage. C57BL/6 and uPAR-KO (*Plaur*^–/–^) mice were purchased from The Jackson Laboratory and the transgenic msuPAR1-OE model, overexpressing full-length suPAR in adipocytes, was generated in-house as recently described (44). All animals were sacrificed for further testing 24 hours after sepsis induction. Methodology of biomarker measurements, kidney Luminex assay, and tissue and flow cytometry analyses (Supplemental Figure 11) are provided in the supplemental material.

### Statistics.

Statistical analyses were performed using SPSS Statistics 28 (IBM), Prism 9 (GraphPad Software), and FlowJo 10.7 Software (BD Life Sciences). Two-sided *P* values of less than 0.05 were considered statistically significant. Continuous variables are presented as mean (±SD); categoric variables are presented as proportions (%). ANOVA test was used for multiple-group comparisons, 2-tailed Student’s *t* test was used for pairwise comparisons, and χ^2^ test for categorical variables. Correlations were assessed by using Pearson’s correlation analysis. Survival differences of mouse strains were evaluated by the Kaplan-Meier method and log-rank testing. deLong’s test was used for comparison of areas under the receiver operating characteristics curves (AUC-ROC). To identify determinants of AKI, we used one logistic regression model with “RRT or death” and MAKE_7_ as binary dependent variables adjusting for age, male sex, CKD, and SCr levels and the presence of septic shock at study inclusion, and another model with the same dependent variables adjusting for age, male sex, CKD, and SOFA score at enrollment (Table 2). To determine additional diagnostic value of suPAR over all kidney biomarkers studied, we utilized 2 models to predict RRT or death, including SCr, NGAL, KIM-1, TIMP-2•IGFBP7, and albuminuria at baseline with and without suPAR at baseline.

### Study approval.

The human studies at Heidelberg University Hospital were approved by the local Ethics Committee of the Medical Faculty of Heidelberg (S-200/2017) and registered at the German Clinical Trials Register (DRKS-ID: DRKS00012446). Written informed consent was obtained from all participants or their legal representatives. All animal experiments were carried out according to NIH *Guide for the Care and Use of Laboratory Animals* (National Academies Press, 2011) and approved by the Rush University (Chicago, Illinois) Institutional Animal Care and Use Committee (protocol 19-014).

## Author contributions

CN and JR conceived the design of the entire study, were responsible for acquisition, analysis, and interpretation of data, and drafted the manuscript. CR and RS assisted with acquisition, analysis, and interpretation of human data and assisted in drafting the manuscript. SSH, CS, FK, MS, FU, FCFS, MOF, EK, JEO, UM, and MZ contributed to the acquisition and interpretation of the clinical data and critically revised the manuscript. CW and EH assisted in the conduct, design, and interpretation of animal experiments. CN planned, conducted, and analyzed the animal experiments. BS, YC, JL, and RR assisted in the preparation, execution, and interpretation of animal experiments. TB, CM, and MAW designed the conception of the human study, assisted with analysis and interpretation of data, and critically revised the manuscript. All authors approved the final version of the manuscript for publication.

## Supplementary Material

Supplemental data

## Figures and Tables

**Table 1:**
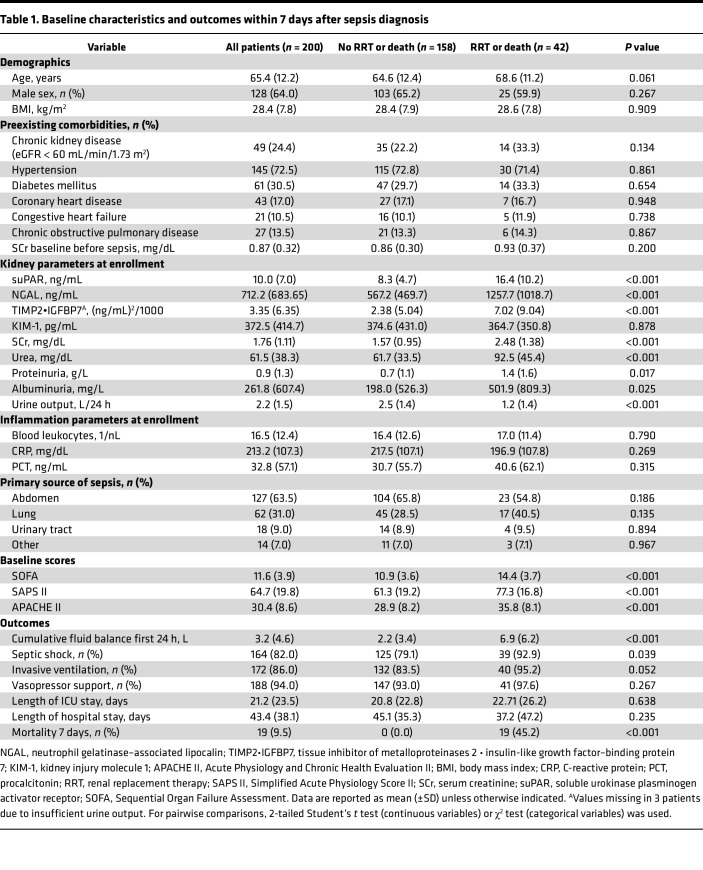
Baseline characteristics and outcomes within 7 days after sepsis diagnosis

**Table 2:**
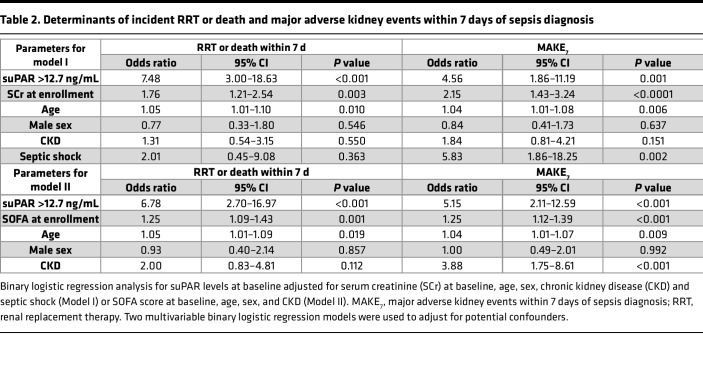
Determinants of incident RRT or death and major adverse kidney events within 7 days of sepsis diagnosis

**Table 3:**
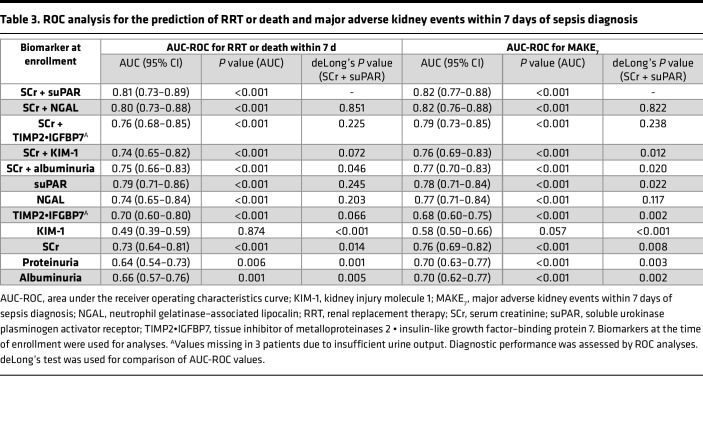
ROC analysis for the prediction of RRT or death and major adverse kidney events within 7 days of sepsis diagnosis

## Version 1 04/10/2023

Electronic publication

## References

[1] Kellum JA, Prowle JR (2018). Paradigms of acute kidney injury in the intensive care setting. Nat Rev Nephrol.

[2] Nusshag C (2017). Issues of acute kidney injury staging and management in sepsis and critical illness: a narrative review. Int J Mol Sci.

[3] Uchino S (2005). Acute renal failure in critically ill patients: a multinational, multicenter study. JAMA.

[4] Kellum JA (2012). Kidney disease: improving global outcomes (KDIGO) acute kidney injury work group. KDIGO clinical practice guideline for acute kidney injury. Kidney Int.

[5] Gaudry S (2021). Comparison of two delayed strategies for renal replacement therapy initiation for severe acute kidney injury (AKIKI 2): a multicentre, open-label, randomised, controlled trial. Lancet.

[6] Gaudry S (2019). Timing of renal replacement therapy for severe acute kidney injury in critically ill patients. Am J Resp Crit Care.

[7] Barbar SD (2018). Timing of renal-replacement therapy in patients with acute kidney injury and sepsis. N Engl J Med.

[8] Gaudry S (2016). Initiation strategies for renal-replacement therapy in the intensive care unit. N Engl J Med.

[9] Ostermann M (2020). Recommendations on acute kidney injury biomarkers from the acute disease quality initiative consensus conference. JAMA Netw Open.

[10] Singer M (2016). The third international consensus definitions for sepsis and septic shock (Sepsis-3). JAMA.

[11] Ergin B (2015). The renal microcirculation in sepsis. Nephrol Dial Transplant.

[12] Hayek SS (2020). Soluble urokinase receptor and acute kidney injury. N Engl J Med.

[13] Azam TU (2020). Soluble urokinase receptor (suPAR) in COVID-19-related AKI. J Am Soc Nephrol.

[14] Nusshag C (2019). Cell cycle biomarkers and soluble urokinase-type plasminogen activator receptor for the prediction of sepsis-induced acute kidney injury requiring renal replacement therapy: a prospective, exploratory study. Crit Care Med.

[15] Backes Y (2012). Usefulness of suPAR as a biological marker in patients with systemic inflammation or infection: a systematic review. Intensive Care Med.

[16] Wei C (2021). suPAR, a circulating kidney disease factor. Front Med (lausanne).

[17] Rasmussen LJH (2021). Soluble urokinase plasminogen activator receptor (suPAR) as a biomarker of systemic chronic inflammation. Front Immunol.

[18] Skalec T (2022). Soluble urokinase-type plasminogen activator receptor levels as a predictor of kidney replacement therapy in septic patients with acute kidney injury: an observational study. J Clin Med.

[19] Mondino A, Blasi F (2004). uPA and uPAR in fibrinolysis, immunity and pathology. Trends Immunol.

[20] Montuori N, Ragno P (2009). Multiple activities of a multifaceted receptor: roles of cleaved and soluble uPAR. Front Biosci (Landmark Ed).

[21] Gussen H (2019). Neutrophils are a main source of circulating suPAR predicting outcome in critical illness. J Intensive Care.

[22] Kiyan Y (2020). TLR4 response to LPS is reinforced by urokinase receptor. Front Immunol.

[23] Koch A (2014). Clinical relevance and cellular source of elevated soluble urokinase plasminogen activator receptor (suPAR) in acute liver failure. Liver Int.

[24] Rovina N (2020). Soluble urokinase plasminogen activator receptor (suPAR) as an early predictor of severe respiratory failure in patients with COVID-19 pneumonia. Crit Care.

[25] Medeiros P (2015). Acute kidney injury in septic patients admitted to emergency clinical room: risk factors and outcome. Clin Exp Nephrol.

[26] Honore PM (2016). Urinary tissue inhibitor of metalloproteinase-2 and insulin-like growth factor-binding protein 7 for risk stratification of acute kidney injury in patients with sepsis. Crit Care Med.

[27] Pickkers P (2021). Acute kidney injury in the critically ill: an updated review on pathophysiology and management. Intensive Care Med.

[28] Titeca-Beauport D (2020). Urine cell cycle arrest biomarkers distinguish poorly between transient and persistent AKI in early septic shock: a prospective, multicenter study. Crit Care.

[29] Klein SJ (2018). Biomarkers for prediction of renal replacement therapy in acute kidney injury: a systematic review and meta-analysis. Intensive Care Med.

[30] Singer E (2013). Neutrophil gelatinase-associated lipocalin: pathophysiology and clinical applications. Acta Physiol (Oxf).

[31] Wheeler DS (2008). Serum neutrophil gelatinase-associated lipocalin (NGAL) as a marker of acute kidney injury in critically ill children with septic shock. Crit Care Med.

[32] Bagshaw SM (2010). Plasma and urine neutrophil gelatinase-associated lipocalin in septic versus non-septic acute kidney injury in critical illness. Intensive Care Med.

[33] Kosaka J (2016). Histopathology of septic acute kidney injury: a systematic review of experimental data. Crit Care Med.

[34] Lee S-Y (2012). Distinct pathophysiologic mechanisms of septic acute kidney injury: role of immune suppression and renal tubular cell apoptosis in murine model of septic acute kidney injury. Crit Care Med.

[35] Lerolle N (2010). Histopathology of septic shock induced acute kidney injury: apoptosis and leukocytic infiltration. Intensive Care Med.

[36] Dellepiane S (2020). T cells and acute kidney injury: a two-way relationship. Front Immunol.

[37] Li Z (2022). The pathogenesis of ischemia-reperfusion induced acute kidney injury depends on renal neutrophil recruitment whereas sepsis-induced AKI does not. Front Immunol.

[38] Cruikshank WW (2000). Interleukin-16. J Leukoc Biol.

[39] Bernstein HB (2006). CD4 expression on activated NK cells: ligation of CD4 induces cytokine expression and cell migration. J Immunol.

[40] Robertson MJ (2002). Role of chemokines in the biology of natural killer cells. J Leukoc Biol.

[41] Wang S (2008). Decreased renal ischemia-reperfusion injury by IL-16 inactivation. Kidney Int.

[42] Furuichi K (2008). Chemokine receptor CCR1 regulates inflammatory cell infiltration after renal ischemia-reperfusion injury. J Immunol.

[43] Fava A (2022). Urine proteomics and renal single-cell transcriptomics implicate interleukin-16 in lupus nephritis. Arthritis Rheumatol.

[44] Wei C (2019). uPAR isoform 2 forms a dimer and induces severe kidney disease in mice. J Clin Invest.

[45] Hayek SS (2015). Soluble urokinase receptor and chronic kidney disease. N Engl J Med.

[46] Mossanen JC (2017). Elevated soluble urokinase plasminogen activator receptor and proenkephalin serum levels predict the development of acute kidney injury after cardiac surgery. Int J Mol Sci.

[47] Yokota N (2003). Contrasting roles for STAT4 and STAT6 signal transduction pathways in murine renal ischemia-reperfusion injury. Am J Physiol Renal Physiol.

[48] Bajwa A (2012). Dendritic cell sphingosine 1-phosphate receptor-3 regulates Th1-Th2 polarity in kidney ischemia-reperfusion injury. J Immunol.

[49] Vignali DAA (2008). How regulatory T cells work. Nat Rev Immunol.

[50] Seder RA, Ahmed R (2003). Similarities and differences in CD4^+^ and CD8^+^ effector and memory T cell generation. Nat Immunol.

[51] Fathabad SG (2020). T lymphocytes in acute kidney injury and repair. Semin Nephrol.

[52] Liu M (2006). A pathophysiologic role for T lymphocytes in murine acute cisplatin nephrotoxicity. J Am Soc Nephrol.

[53] Burne-Taney MJ (2003). Acute renal failure after whole body ischemia is characterized by inflammation and T cell-mediated injury. Am J Physiol Renal Physiol.

[54] Rabb H (2000). Pathophysiological role of T lymphocytes in renal ischemia-reperfusion injury in mice. Am J Physiol Renal Physiol.

[55] Kinsey GR, Okusa MD (2014). Expanding role of T cells in acute kidney injury. Curr Opin Nephrol Hypertens.

[56] Godfrey DI (2010). Raising the NKT cell family. Nat Immunol.

[57] Godfrey DI, Kronenberg M (2004). Going both ways: immune regulation via CD1d-dependent NKT cells. J Clin Invest.

[58] Rossjohn J (2012). Recognition of CD1d-restricted antigens by natural killer T cells. Nat Rev Immunol.

[59] Brigl M (2003). Mechanism of CD1d-restricted natural killer T cell activation during microbial infection. Nat Immunol.

[60] Uchida T (2018). Activated natural killer T cells in mice induce acute kidney injury with hematuria through possibly common mechanisms shared by human CD56^+^ T cells. Am J Physiol Renal Physiol.

[61] Kitching AR, Holdsworth SR (2011). The emergence of TH17 cells as effectors of renal injury. J Am Soc Nephrol.

[62] Starr ME (2014). A new cecal slurry preparation protocol with improved long-term reproducibility for animal models of sepsis. PLoS One.

[63] Rhodes A (2017). Surviving sepsis campaign: international guidelines for management of sepsis and septic shock: 2016. Intensive Care Med.

