# Supplementary material for: suPAR links a dysregulated immune response to tissue inflammation and sepsis-induced acute kidney injury
Source: JCI Insight. 2023 Apr 10;8(7):e165740. doi: 10.1172/jci.insight.165740 (PMC10132159; doi:10.1172/jci.insight.165740)
Supplement: Supplemental data [file jciinsight-8-165740-s021.pdf]

Supplemental Data – Table of contents

Supplemental methods

Human studies:

Study design, sample collection and laboratory methods 3-4

Inclusion criteria for potential study participants 4

Exclusion criteria for potential study participants 4

Definitions of baseline serum creatinine 5

Definition of transient and persistent acute kidney injury 5

Criteria for the initiation of renal replacement therapy 5

Animal studies:

Blood collection, tissue harvesting and kidney digestion 6

Measurement of kidney function parameters, suPAR and interleukin 6 6

Kidney luminex assay 7

Flow cytometry staining and analysis 8

Histology, kidney immunofluorescence and TUNEL assay 9

Supplemental figure and figure legends 10-19

Supplemental Figure 1: Knockout of urokinase plasminogen activator receptor protects from sepsis-induced acute kidney injury

Supplemental Figure 2: Elevated blood levels of soluble urokinase plasminogen activator receptor are associated with increased cellular apoptosis in kidney tissue after 24h of sepsis

Supplemental Figure 3: Blood levels of interleukin-6 and soluble urokinase plasminogen activator receptor after sepsis induction in different mouse strains

Supplemental Figure 4: Overview of strain-dependent characterization of leukocyte subsets by flow cytometry after 24h of sepsis induction via i.p. injection of 250µL cecal slurry and untreated mice.

Supplemental Figure 5: Kidney function impairment and extent of increased levels of soluble urokinase plasminogen activator receptor correlate with frequencies of total kidney T cell subsets but not neutrophils in C57BL/6 wild-type after 24h of sepsis

Supplemental Figure 6: Luminex assay of kidney tissue from untreated mice strains reveals local upregulation of interleukine-16 and C-C motif chemokine ligand 3, and downregulation of thrombospondin 4 in transgenic mice with overexpression of soluble urokinase plasminogen activator receptor.

Supplemental Figure 7: Sustained elevation of blood soluble urokinase plasminogen activator receptor levels is linked to increased accumulation of $CD4^+$ and $CD8^+$ T cells in kidney cortex and medulla of CS-treated transgenic C57BL/6 with overexpression of soluble urokinase plasminogen activator receptor.

Supplemental Figure 8: Sustained elevation of blood soluble urokinase plasminogen activator receptor levels is linked to increased accumulation of $CD4^+$ and $CD8^+$ T cells in kidney cortex and medulla of untreated transgenic C57BL/6 with overexpression of soluble urokinase plasminogen activator receptor.

Supplemental Figure 9: Soluble urokinase plasminogen activator receptor levels do not affect the amount of Ly6G/C^+ cells in kidney tissue of C57BL/6 wild-type, urokinase plasminogen activator receptor knockout and transgenic C57BL/6 with overexpression of soluble urokinase plasminogen activator receptor after 24h of sepsis and in untreated mice.

Supplemental Figure 10: Increasing volumes of intraperitoneal cecal slurry are associated with increased blood levels of soluble urokinase plasminogen activator receptor, progressive impairment of kidney function and poor survival.

Supplemental Figure 11: Gating strategies for kidney single cell suspensions labeled with fluorophore-conjugated anti-mouse antibodies

Supplemental Tables 20-22

Supplemental Table 1: Receiver-operating characteristic analysis for the prediction of renal replacement therapy within 7 days of sepsis diagnosis

Supplemental Table 2: Receiver-operating characteristic analysis for the prediction of “death” within 7 days of sepsis diagnosis

References 23

Supplemental methods

Human studies

PredARRT-Sep-Trial: study design, sample collection and laboratory methods

The PredARRT-Sep-Trial (**Pred**iction of **A**KI with the need for **R**enal **R**eplacement **T**herapy by the use of biomarkers in patients with **Sep**sis or septic shock) is a prospective observational study designed to evaluate and develop new innovative kidney damage and function biomarker for improved kidney outcome prediction and future AKI management. Restrictive criteria for renal replacement therapy (RRT) were predefined to allow sufficient time for autonomous renal recovery and to enable standardized biomarker comparison. The primary outcome was RRT within 7 days of sepsis diagnosis. The first 100 patients of this trial were published in Critical Care Medicine with a dominant focus on urinary cell cycle arrest biomarkers (1). The PredARRT-Sep-Trial was extended to 200 patients to allow for more meaningful and longitudinal assessment of kidney biomarkers on a larger scale. Now, for the first presentation of 200 patients, we combined RRT or death within 7 days of sepsis as main outcome measure to avoid excluding the sickest patients from our primary analyses. Of 1620 patients assessed for eligibility, 200 patients met the inclusion criteria and were enrolled in the study between May 2017 and September 2019. Additional outcomes were RRT within 7 days, 7-day mortality, transient/persistent AKI or major adverse kidney events ($MAKE_7$: combinatory endpoint of RRT, death or persistent AKI) within 7 days of sepsis diagnosis and 30-d all-cause mortality.

Sequential blood and urine samples were collected over a 7-day period after sepsis diagnosis / study enrollment. We obtained serum and plasma samples at the time of enrollment and 12h, 24h, 48h, 72h (3d), 96h (4d), 120h (5d) and 168h (7d) later, and urine samples at the time of enrollment, 12h, 24h, 36h, 48h, 60h, 72h (3d), 96h (4d), 120h (5d) and 168h (7d). Urine and blood samples were centrifuged at 3,000 rounds per minute (rpm) for 10 and 15 minutes, respectively. The supernatants were immediately transferred to Eppendorf tubes and stored at -

80°C. All samples were thawed directly prior to analysis. Serum levels of suPAR, NGAL and KIM-1 were measured by experienced, external technicians (for suPAR at ViroGates Birkerød, Denmark) who were blinded to the clinical data, using a commercially available ELISA (suPARnostic assay by ViroGates, Birkerød, Denmark; human NGAL by Bioporto Diagnostics, Hellerup, Denmark; human KIM-1 by R&D Systems, Minneapolis, USA). TIMP2 and IGFBP7 were measured using a commercially available, standardized point-of-care assay (NephroCheck, Astute Medical, San Diego, CA, USA). Test results are given as product of both markers in $(ng/ml)^2$/ 1000. All other laboratory values were measured in the accredited Central Laboratory of Heidelberg University Hospital. Clinical standard data were gathered. Urine output, routine blood results, and vital parameters were collected daily over a 7-day period. The severity of kidney function impairment was assessed by SCr, and standard urinary parameters. Additional physiological and clinical information was obtained by calculating the Sequential Organ Failure Assessment (SOFA) score, the Simplified Acute Physiology Score II (SAPS II) and the Acute Physiology and Chronic Health Evaluation II (APACHE II) score.

Inclusion criteria for potential study participants

(1) Fulfilment of Sepsis-3 criteria irrespective of actual kidney function (judged by clinical experts)
(2) Age ≥ 18 years

Exclusion criteria for potential study participants

(3) Refusal to participate
(4) Pre-existing RRT dependency or immediate need for RRT at the time of ICU admission
(5) Decompensated liver cirrhosis (hepatorenal syndrome)
(6) No urinary catheter
(7) Life expectancy shorter than 24h.

Definitions of baseline serum creatinine for AKI staging [*] ***in descending order***

(1) Most recent value within 7 days prior to hospital admission or elective surgery

(2) or nadir value within 7 days prior to hospital admission or elective surgery

(3) or SCr value closest to enrolment.

Definition of transient and persistent acute kidney injury

(1) Transient acute kidney injury: SCr decrease of >33% or return to baseline within 72h in the absence of oliguria and anuria (as recently suggested by Duff et al. (2))

(2) Persistent acute kidney injury: no SCr decrease of >33% or return to baseline or manifest oliguria and anuria > 72h

Predefined, restrictive criteria for the initiation of renal replacement therapy (derived from Gaudry et al (3)):

Urea >240 mg/dL, serum potassium >6 mmol/L or >5.5 despite treatment, pH <7.15 in the context of pure metabolic acidosis or mixed acidosis (PaCO2 of 50 mmHg or more without the possibility of increasing alveolar ventilation), acute pulmonary oedema due to fluid overload requiring >5l O_2 to maintain a peripheral capillary oxygen saturation (SpO_2) > 95% or a fraction of inspired oxygen (FiO_2) >50%.

Animal studies

All animal studies were conducted at the Reiser Lab in Chicago and approved by the Rush University Institutional Animal Care and Use Committee (IACUC) protocol, #19-014.

Blood collection, tissue harvesting and kidney digestion

Mice were anesthetized using Ketamine/Xylazine before each blood collection. Blood collection was performed via orbital venous puncture. Collected blood was allowed to clot at room temperature for 2 h before centrifugation (at 5,000 RPM for 5min) to collect sera for analyses. Sera were stored at −80°C until analyses. After 24 hours of sepsis, mice were anesthetized and sacrificed while kidneys were perfused via cardiac puncture with cold PBS. Kidneys were immediately prepared for further analysis. The same methods were applied in untreated mice. Kidney tissue was manually dissected, minced, and digested in 5 mL Hank's Balanced Salt Solution (HBSS) containing 0.2 mg/mL Liberase (Roche) and 100 U/mL DNase I (Sigma), for 50 min at 37°C with gentle shaking. The digestion was stopped by adding 10 mL PBS containing 0.5% bovine serum albumin (BSA) and 2 mM EDTA. Kidney tissues were then filtered through a 70µm nylon mesh and subsequently red blood cells (RBC) were lysed by incubating with RBC lysis buffer containing ammonium chloride for 5min (Biolegend). Single cell suspensions were stained for flow cytometry.

Measurement of kidney function parameters, suPAR and interleukin 6

QuantiChromTM Urea Assay Kit (BioAssay Systems, Catalog # DIUR-100), Creatinine Assay KIT (Sigma Aldrich, Catalog #MAK080), Mouse uPAR DouSet ELISA (R&D Systems, Catalog # DY008) and Mouse IL-6 Quantikine ELISA KIT (R&D Systems, Catalog # M600B) were used for the measurement of blood urea, creatinine, suPAR and IL-6 levels. All assays were performed according to the manufacturer's instructions.

Kidney luminex assay

We performed mouse premixed multiplex magnetic Luminex assays (R&D Systems) following the manufacturer's instruction. The following analytes were included for analysis: CCL2, CCL3, CCL4, CXCL2, CXCL10, FGF-21, GM-CSF, IFN-gamma, IGFBP3, IL-1 beta, IL-2, IL-4, IL-5, IL-6, IL-10, IL-12 p70, IL-13, IL-16, IL-17, IL-25, IL-27, IL-33, MMP-9, podocalyxin, periostin, PCSK9, renin, TIM-1/KIM-1, thrombospondin-4, TNF-alpha and uPAR. In brief, non-treated mice (n=6 for WT and n=4 for suPAR-overexpressing mice) were sacrificed with both kidneys taken out. The kidneys were cut in halves, with one half used for Luminex assays. The kidney tissues were homogenized in cell lysis buffer (R&D Systems) and the total protein concentration was measured by BCA assay (Biorad). Equal volume of standards and samples (50 µl) was loaded into the plate for incubation. After final wash, data were acquired using Luminex FLEXMAP 3D analyzer (Luminex). Final values (net mean fluorescent intensity) were corrected against total protein amount loaded.

Flow cytometry staining and analysis

Kidney single cell suspensions (2x10^6 cells in 100 µl total volume) were incubated with Aqua Live Dead fixable stain (Thermo Fisher Scientific, Rockford, IL), FcR-blocking reagent (BD Biosciences, San Jose, CA) and labeled with fluorophore-conjugated anti-mouse antibodies at dilutions following manufacturer recommendations. Red blood cell lysis was performed with ammonium chloride. Heparin blood was stained with fluorophore-conjugated anti-mouse antibodies following manufacturer recommendations and red blood cell lysis was performed using FACS lysis solution (BD) according to the manufacturer's instructions. Data were acquired using an LSR-Fortessa flow cytometer and analyzed using FlowJo software version 10.7. The gating strategies are displayed in Supplemental Figure 11. Fluorophore-conjugated antibodies were used directed to CD45, CD3, CD4, CD8, CD19, NK1.1, Ly6G, Ly6C, F4/80, CD11b, CD11c.

Antibody	Clone	Fluorochrome	Source	Catalog #
anti-mouse CD45	30-F11	PE	BioLegend	103106
anti-mouse CD3	17A2	PerCP/Cy5.5	BioLegend	100217
anti-mouse CD4	RM4-5	PE/Cy7	BioLegend	100528
anti-mouse CD8	53-6.7	Alexa Fluor 700	BioLegend	100730
anti-mouse CD19	6D5	Brilliant Violet 421	BioLegend	115549
anti-mouse NK-1.1	PK136	FITC	BioLegend	108706
anti-mouse Ly-6G	1A8	PE/Cy7	BioLegend	127617
anti-mouse Ly-6C	HK1.4	Brilliant Violet 605	BioLegend	128036
anti-mouse F4/80	BM8	APC	BioLegend	123116
anti-mouse CD11b	M1/70	Alexa Fluor 488	BioLegend	101219
anti-mouse CD11c	N418	Brilliant Violet 785	BioLegend	117335

Histology, kidney immunofluorescence and TUNEL assay

Tissues for standard histology were fixed in 10% formalin overnight while shaking. Tissues were then dehydrated in increasing grades of ethanol and xylene, embedded in paraffin, and cut into 7-µm sections. Hematoxylin and eosin staining was performed at the University of Illinois at Chicago Histology Core. Histologic analysis was performed blinded to the study group. Immunofluorescence staining was performed in-house. Frozen mouse kidney tissues were cut at 4 µm thickness and fixed with cold acetone for 5 minutes. After blocking with 1% bovine serum albumin (BSA) in phosphate buffered saline (PBS), samples were stained with the respective primary antibodies. Rat anti-mouse antibodies for CD4 (Invitrogen, GK1.5, Catalog # 14-0041-85), CD8 (Invitrogen, 53-6.7, Catalog # 14-0081-82) and Ly6G/C (Invitrogen, RB6-8C5, Catalog # 14-5931-82) were used to stain for immune cells (1:50). Rabbit anti-mouse antibody for podocin (SigmaAldrich, Catalog # P0372) was used to stain for glomerula (1:100). The secondary antibodies for CD4, CD8 and Ly6G/C labelling were Alexa Fluor 546–conjugated goat anti-rat IgG (1:1,000; Invitrogen, Catalog # A-11081) and Alexa Fluor 488–conjugated goat anti-rabbit IgG (1:500; Invitrogen, Catalog # A-11008) for podocin. After sufficient washing, stained samples were mounted with ProLong Gold Antifade Reagent with DAPI (Molecular Probes, P36935). Spleen sections with primary and secondary antibodies and secondary antibodies only served as controls. For quantification of apoptosis, an in situ BrdU-Red DNA fragmentation (TUNEL) assay was used (Abcam, Catalog #, ab66110). Frozen kidney tissue sections were fixed in 4% paraformaldehyde/PBS and processed according to the manufacturer's instructions. Instead of proteinase K, tissue sections were permeabilized with 0,5% Triton/PBS for 20 minutes. Pretreatment of kidney tissue sections from untreated C57BL/6 mice with DNAse served as positive control. Images were obtained and analyzed using a LSM 700 confocal microscope (Carl Zeiss).

Supplemental figures and figure legends

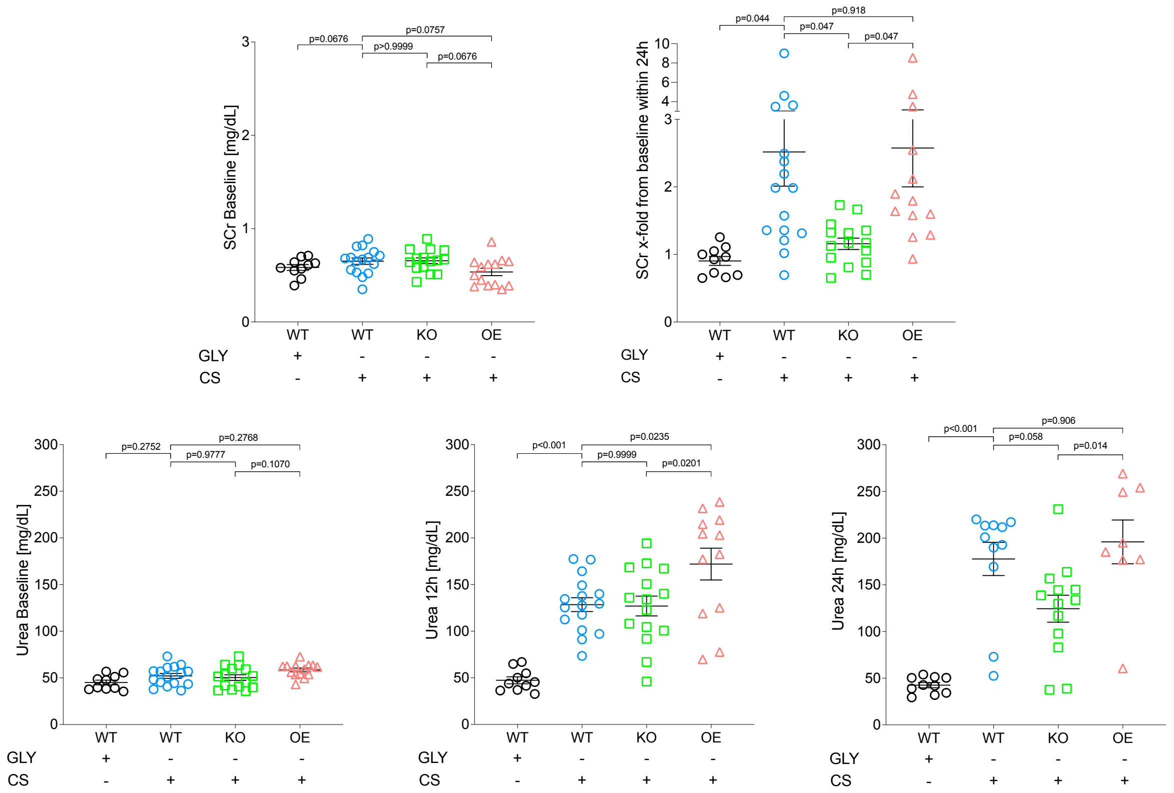

Supplemental Figure 1: Knockout of urokinase plasminogen activator receptor protects from sepsis-induced acute kidney injury. Kidney function biomarkers within 24h after sepsis induction in C57BL/6 wild-type (WT, n=16), urokinase plasminogen activator receptor knockout (KO, n=15) and transgenic mice with overexpression of soluble urokinase plasminogen activator receptor (OE, n=14). Sepsis was induced via i.p. injection of 250 µL cecal slurry (CS). 15% glycerol (GLY) served as control (vehicle control). Data are reported as mean (standard error of the mean [SEM]). One-way ANOVA test was used for group comparisons.

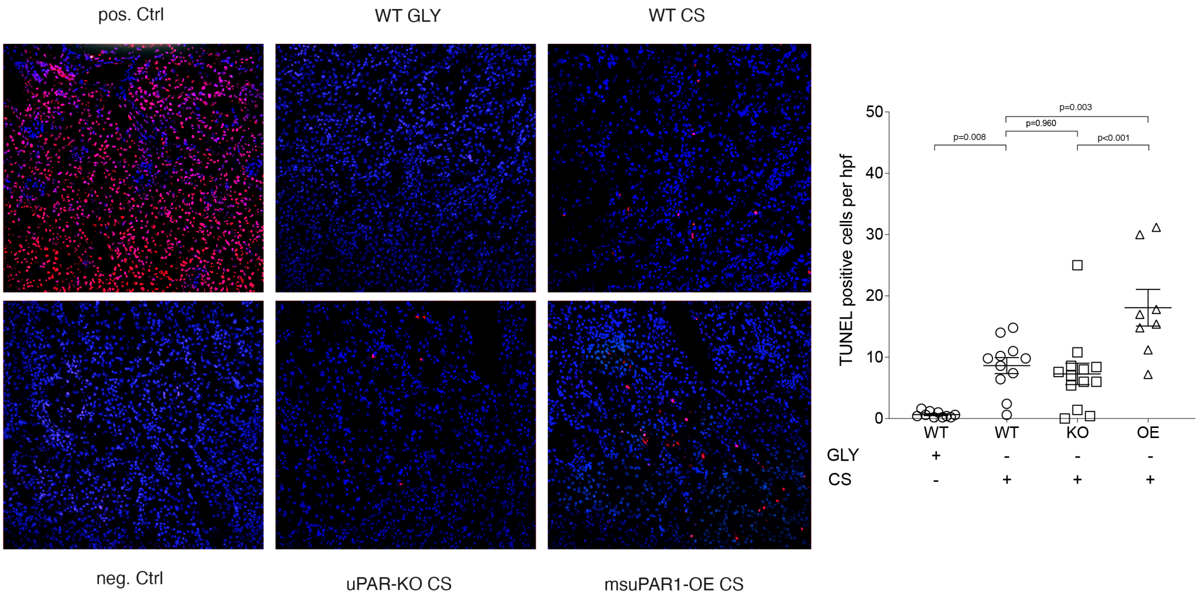

Supplemental Figure 2: Elevated blood levels of soluble urokinase plasminogen activator receptor are associated with increased cellular apoptosis in kidney tissue after 24h of sepsis. DNA fragmentation in apoptosis was evaluated by BrdU-Red DNA fragmentation (TUNEL) assay, TUNEL positive cells (red). Nuclei were stained with 4′,6-diamidino-2-phenylindole (blue). TUNEL positive cell number was determined from 10 representative high-power fields (hpf) per animal. Tissue from untreated C57BL/6 wildtype served as negative control (neg. Ctrl). The same tissue treated with 0.5% Triton and DNAse served as positive control (pos. Ctrl). Sepsis was induced via i.p. injection of 250 µL cecal slurry (CS) in C57BL/6 wild-type (WT), urokinase plasminogen activator receptor (uPAR) knockout (KO) and transgenic C57BL/6 with overexpression of soluble urokinase plasminogen activator receptor (OE). 15% glycerol (GLY) served as control (vehicle control). Data are reported as mean (SEM). One-way ANOVA test was used for group comparisons.

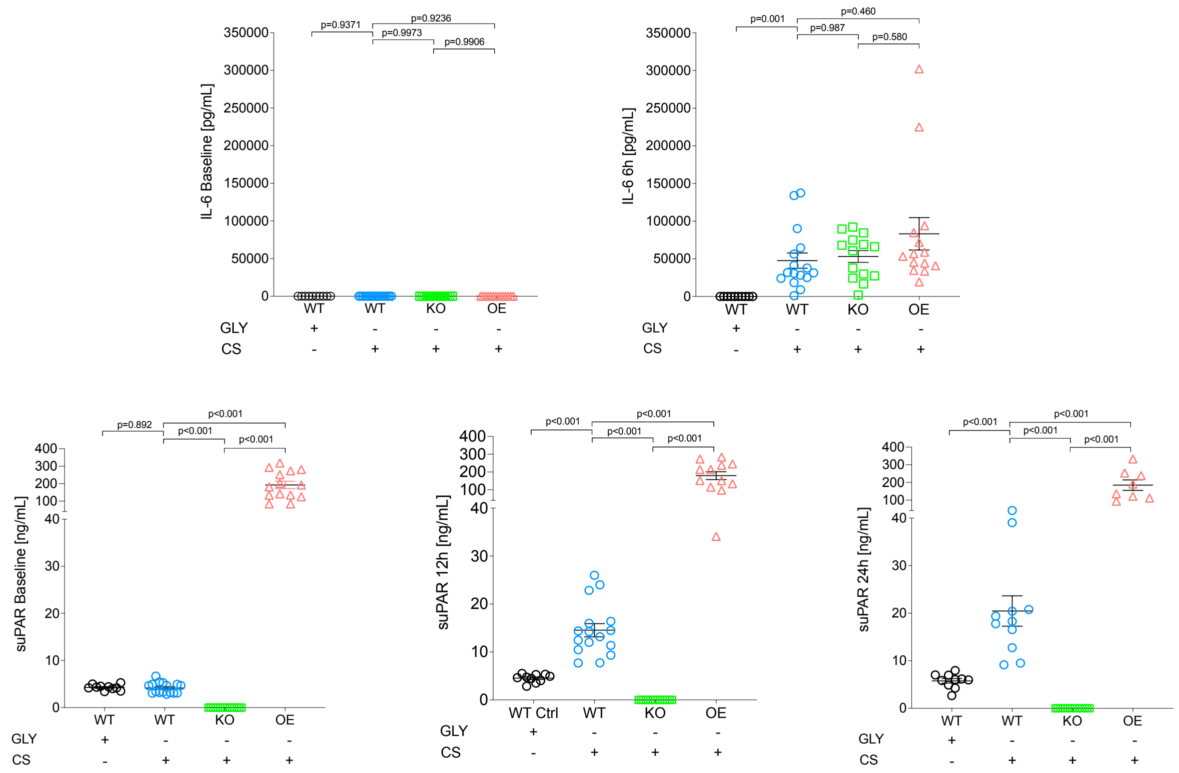

Supplemental Figure 3: **Blood levels of interleukin-6 (IL-6) and soluble urokinase plasminogen activator receptor after sepsis induction in different mouse strains.** Sepsis was induced via i.p. injection of 250 µL cecal slurry (CS) in C57BL/6 wild-type (WT, n=16), urokinase plasminogen activator receptor (uPAR) knockout (KO, n=15) and transgenic C57BL/6 with overexpression of soluble urokinase plasminogen activator receptor (OE, n=14). 15% glycerol (GLY) served as control in WT (vehicle control, n=10). Data are reported as mean (SEM). One-way ANOVA test was used for group comparisons.

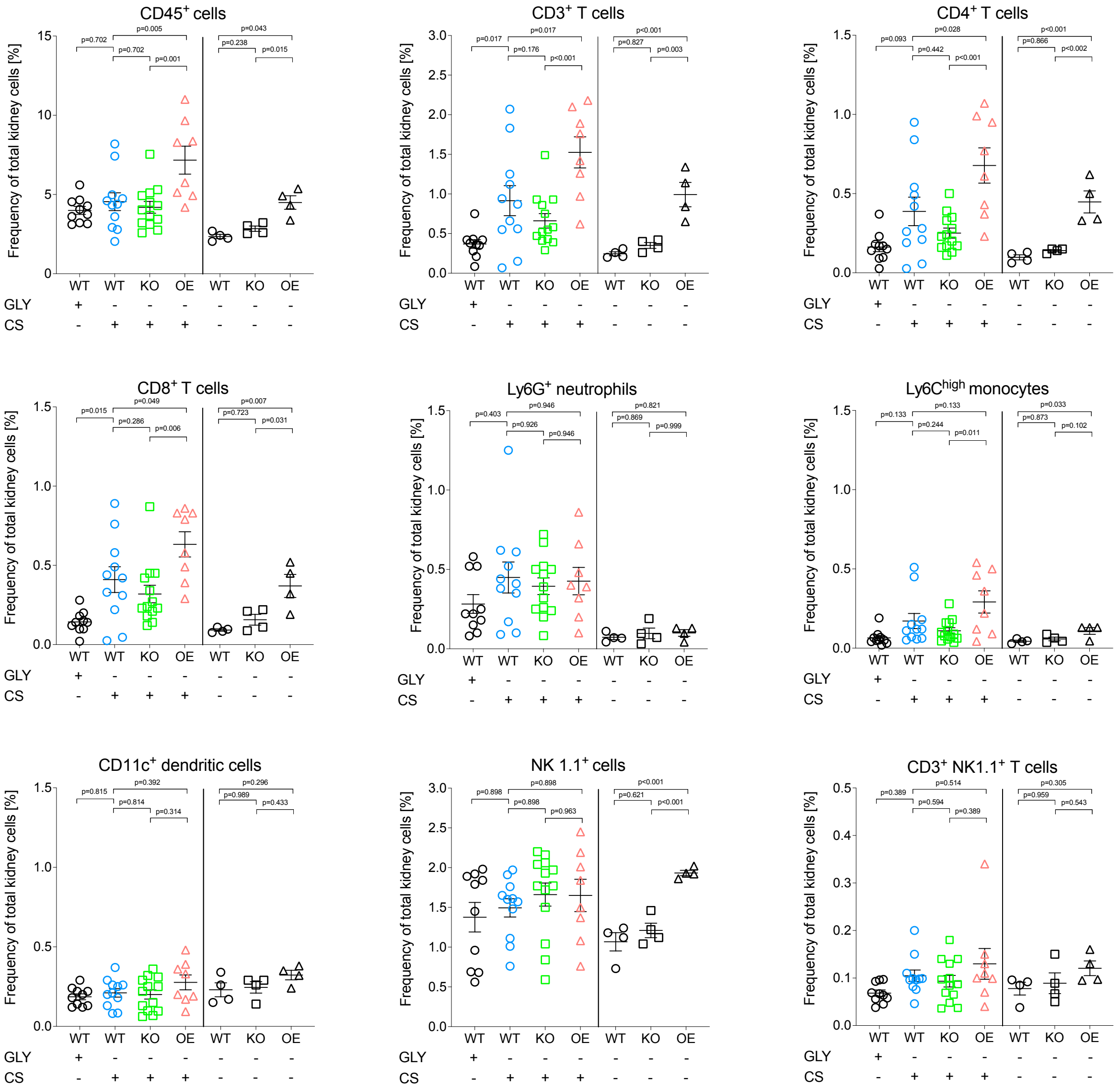

Supplemental Figure 4: Overview of strain-dependent characterization of leukocyte subsets by flow cytometry after 24h of sepsis induction (left part) via i.p. injection of 250µL cecal slurry (CS) and untreated mice (right part). Injection of 15% glycerol (GLY) served as control (vehicle solution). WT C57BL/6 wild-type, KO urokinase plasminogen activator receptor knockout, OE transgenic C57BL/6 with overexpression of soluble urokinase plasminogen activator receptor. One-way ANOVA test was used for group comparisons.

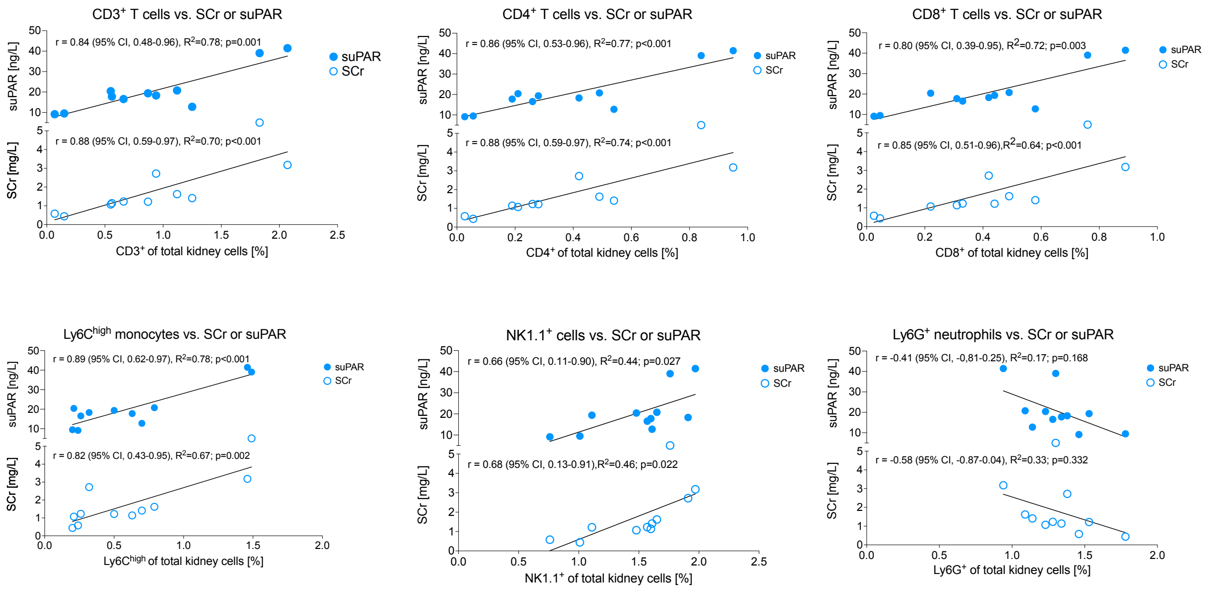

Supplemental Figure 5: Kidney function impairment and extent of increased levels of soluble urokinase plasminogen activator receptor (suPAR) correlate with frequencies of total kidney T cell subsets but not neutrophils in C57BL/6 wild-type after 24h of sepsis. Pearson correlation analysis of kidney immune cells with serum creatinine (SCr) and blood levels of soluble urokinase plasminogen activator receptor in C57BL/6 wild-type. Correlations were assessed by using Pearson's correlation analysis.

	GM-CSF	TSP4 *	TNFα	IL-12	MCP-1	IL-1β	CXCL2	IL-2	IL-4	IL-5	IL-6	IL-10	IL-13
WT	1	1	1	1	1	1	1	1	1	1	1	1	1
OE	1.02	0.56	0.79	0.92	0.32	0.85	0.79	0.81	0.89	0.96	1.00	0.90	0.87

	IL-17	IFNγ	IL-16 *	CXCL10	IL-33	CCL3 *	CCL4	IL-25	IL-27	FGF-21	MMP-9	IGFBP3	uPAR
WT	1	1	1	1	1	1	1	1	1	1	1	1	1
OE	0.81	0.72	1.30	1.11	0.90	1.99	0.54	0.99	0.90	0.85	1.31	1.07	0.89

Supplemental Figure 6: Luminex assay of kidney tissue from untreated mice strains reveals local upregulation of interleukine-16 and C-C motif chemokine ligand 3, and downregulation of thrombospondin 4 in transgenic mice with overexpression of soluble urokinase plasminogen activator receptor. WT C57BL/6 wild-type (n=12), OE transgenic C57BL/6 with overexpression of soluble urokinase plasminogen activator receptor (n=8). CCL3 = C-C motif chemokine ligand 3, CCL4 = C-C motif chemokine ligand 4, CXCL2 = C-X-C motif chemokine ligand 2, CXCL10 = C-X-C motif chemokine ligand 10, FGF-21 = Fibroblast growth factor-21, GM-CSF = Granulocyte-macrophage colony-stimulating factor, IFNγ = Interferon gamma, IGFBP3 = Insulin-like growth factor-binding protein 3, IL-1β = Interleukin-1 beta, IL-2 = Interleukin-2, IL-4 = Interleukin-4, IL-5 = Interleukin-5, IL-6 = Interleukin-6, IL-10 = Interleukin-10, IL-12 = Interleukin-12, IL-13 = Interleukin-13, IL-16 = Interleukin-16, IL-17 = Interleukin-17, IL-25 = Interleukin-25, IL-27 = Interleukin-27, IL-33 = Interleukin 33, MCP-1 = monocyte chemoattractant protein-1, MMP-9 = Matrix metallopeptidase-9, TNFα = Tumor necrosis factor alpha, TSP4 = Thrombospondin 4, uPAR = urokinase plasminogen activator receptor. Mean cytokine levels (MFI) in OE are expressed as fold changes relative to mean cytokine levels (MFI) in WT. * Indicates significant difference of OE compared to WT. Two-tailed Student's t test was used for pairwise comparisons.

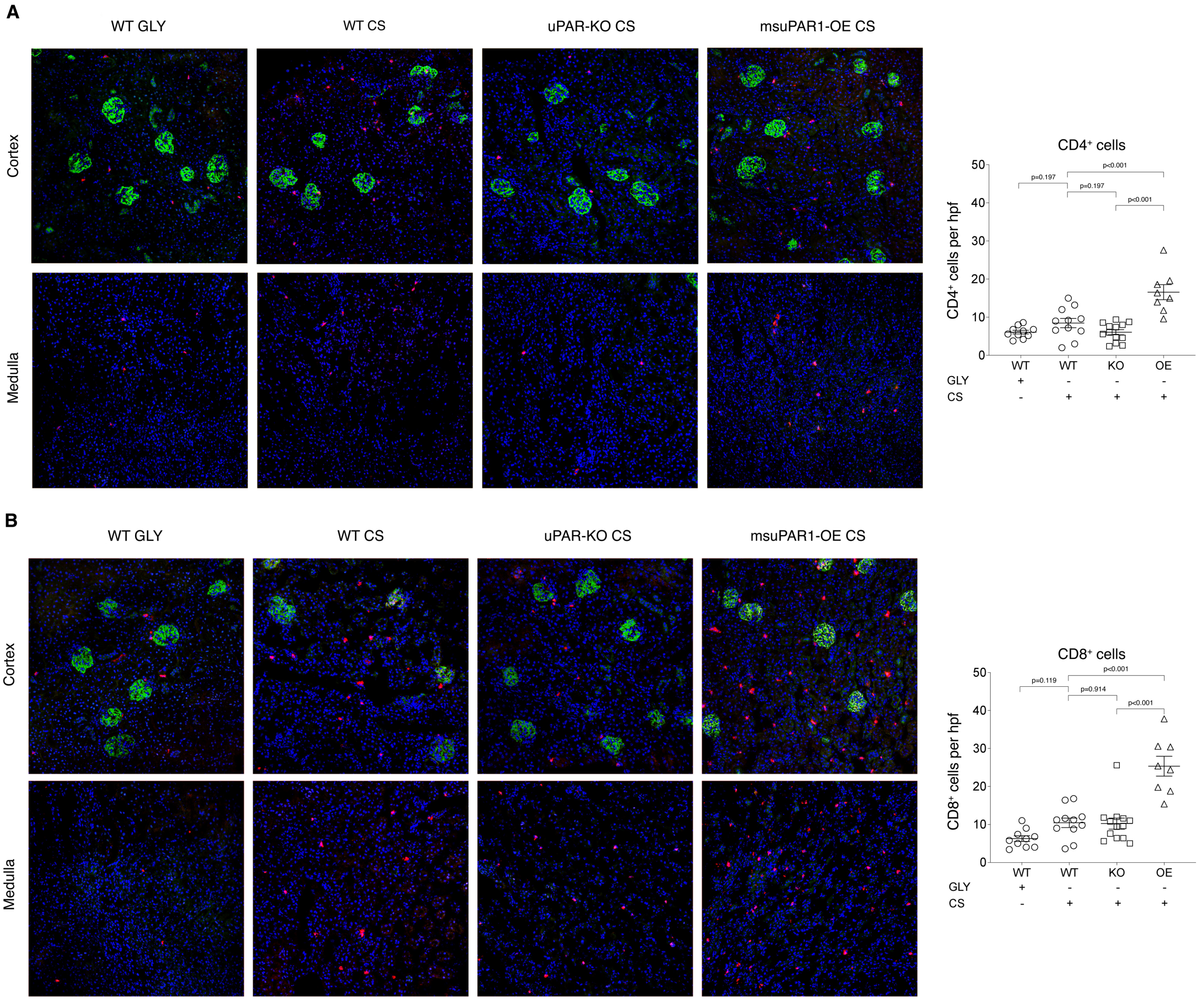

Supplemental Figure 7: Sustained elevation of blood soluble urokinase plasminogen activator receptor levels is linked to increased accumulation of (A) $CD4^+$ and (B) $CD8^+$ T cells in kidney cortex and medulla of CS-treated transgenic C57BL/6 with overexpression of soluble urokinase plasminogen activator receptor. Double immunofluorescence staining for podocin (green) and $CD4^+$ or $CD8^+$ T cells (red) of kidney tissue from C57BL/6 wild-type (WT), urokinase plasminogen activator receptor (uPAR) knockout (KO) and transgenic C57BL/6 with overexpression of soluble urokinase plasminogen activator receptor (OE). Nuclei were stained with 4′,6-diamidino-2-phenylindole (blue). To quantify kidney immune cell accumulation the mean cell number was determined from 10 representative high-power fields (hpf) per animal. Spleen tissue served as positive (primary & secondary antibody) and negative (secondary antibody only) control (data not shown). One-way ANOVA test was used for group comparisons.

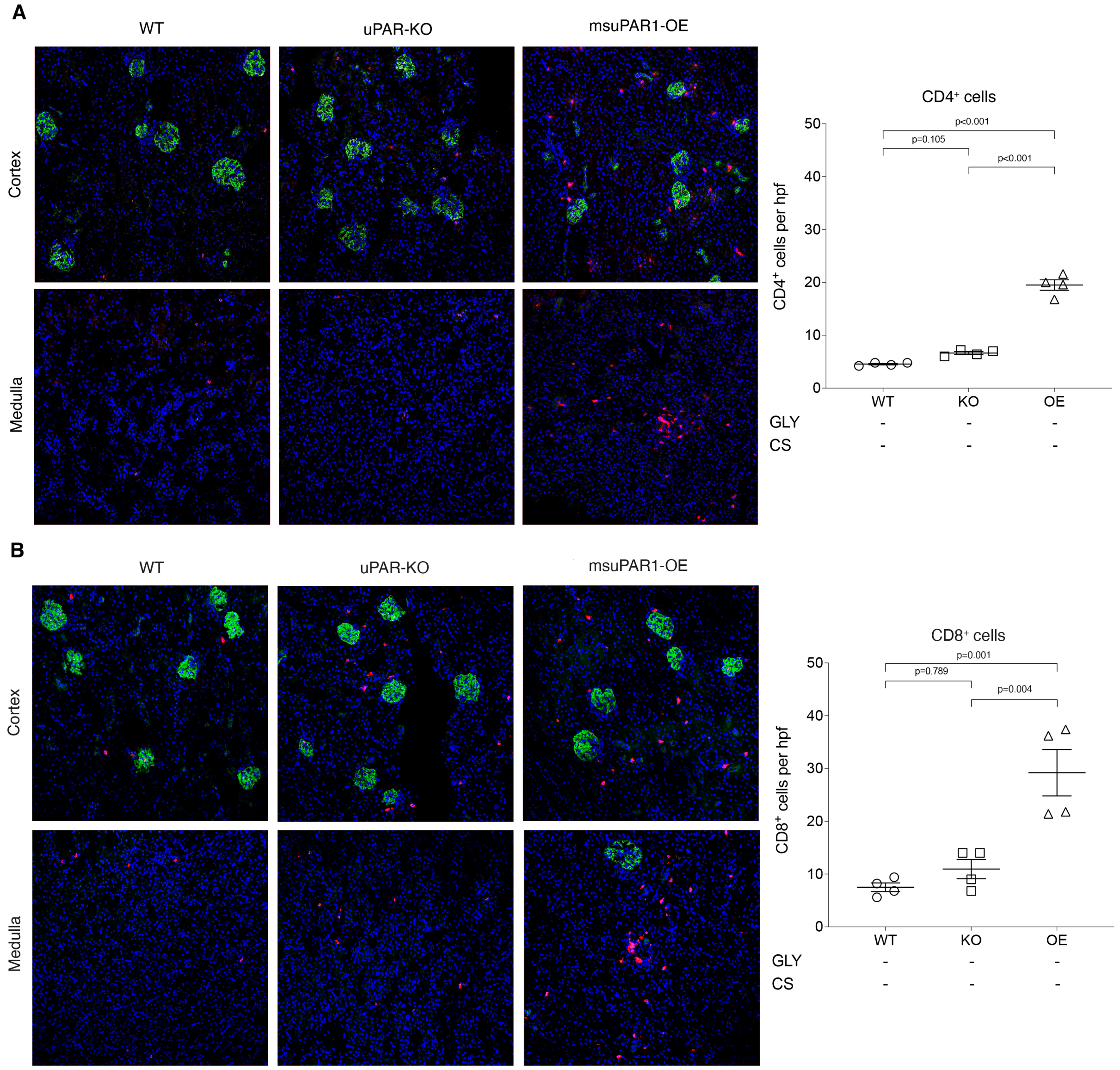

Supplemental Figure 8: Sustained elevation of blood soluble urokinase plasminogen activator receptor levels is linked to increased accumulation of (A) CD4$^+$ and (B) CD8$^+$ T cells in kidney cortex and medulla of untreated transgenic C57BL/6 with overexpression of soluble urokinase plasminogen activator receptor. Double immunofluorescence staining for podocin (green) and CD4+ or CD8+ T cells (red) of kidney tissue from C57BL/6 wild-type (WT), urokinase plasminogen activator receptor (uPAR) knockout (KO) and transgenic C57BL/6 with overexpression of soluble urokinase plasminogen activator receptor (OE). Nuclei were stained with 4′,6-diamidino-2-phenylindole (blue). To quantify kidney immune cell accumulation the mean cell number was determined from 10 representative high-power fields (hpf) per animal. Spleen tissue served as positive (primary & secondary antibody) and negative (secondary antibody only) control (data not shown). One-way ANOVA test was used for group comparisons.

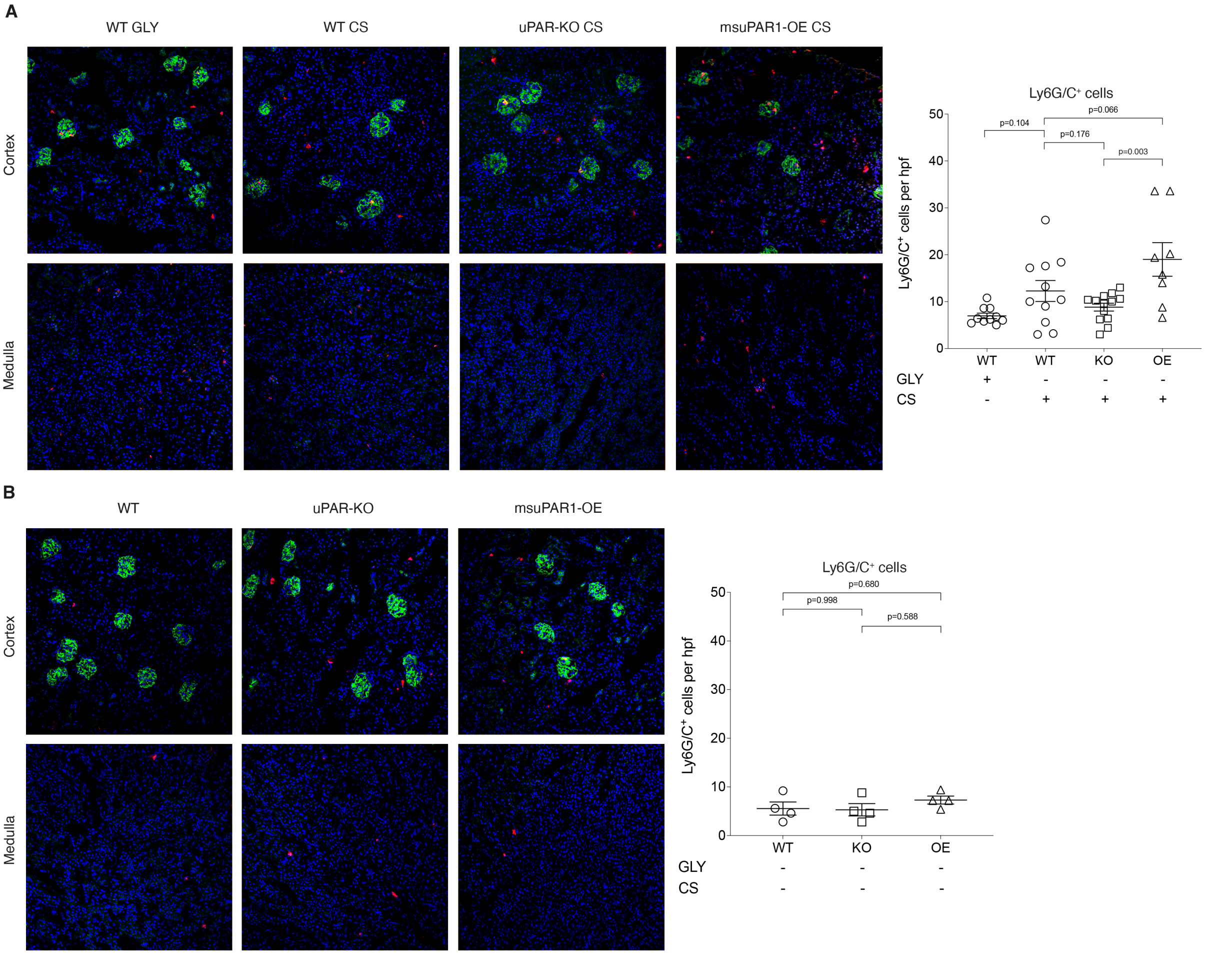

Supplemental Figure 9: Soluble urokinase plasminogen activator receptor levels do not affect the amount of Ly6G/C^{+} cells in kidney tissue of C57BL/6 wild-type (WT), urokinase plasminogen activator receptor (uPAR) knockout (KO) and transgenic C57BL/6 with overexpression of soluble urokinase plasminogen activator receptor (msuPAR1-OE) (A) after 24h of sepsis and (B) in untreated mice. Double immunofluorescence staining for podocin (green) and Ly6G/C^{+} cells (red). Nuclei were stained with 4′,6-diamidino-2-phenylindole (blue). To quantify kidney immune cell aggregation, the mean cell number was determined from 10 representative high-power fields (hpf) per animal. Sepsis was induced via i.p. injection of 250 µL cecal slurry (CS). Spleen tissue served as positive (primary & secondary antibody) and negative (secondary antibody only) control (data not shown). One-way ANOVA test was used for group comparisons.

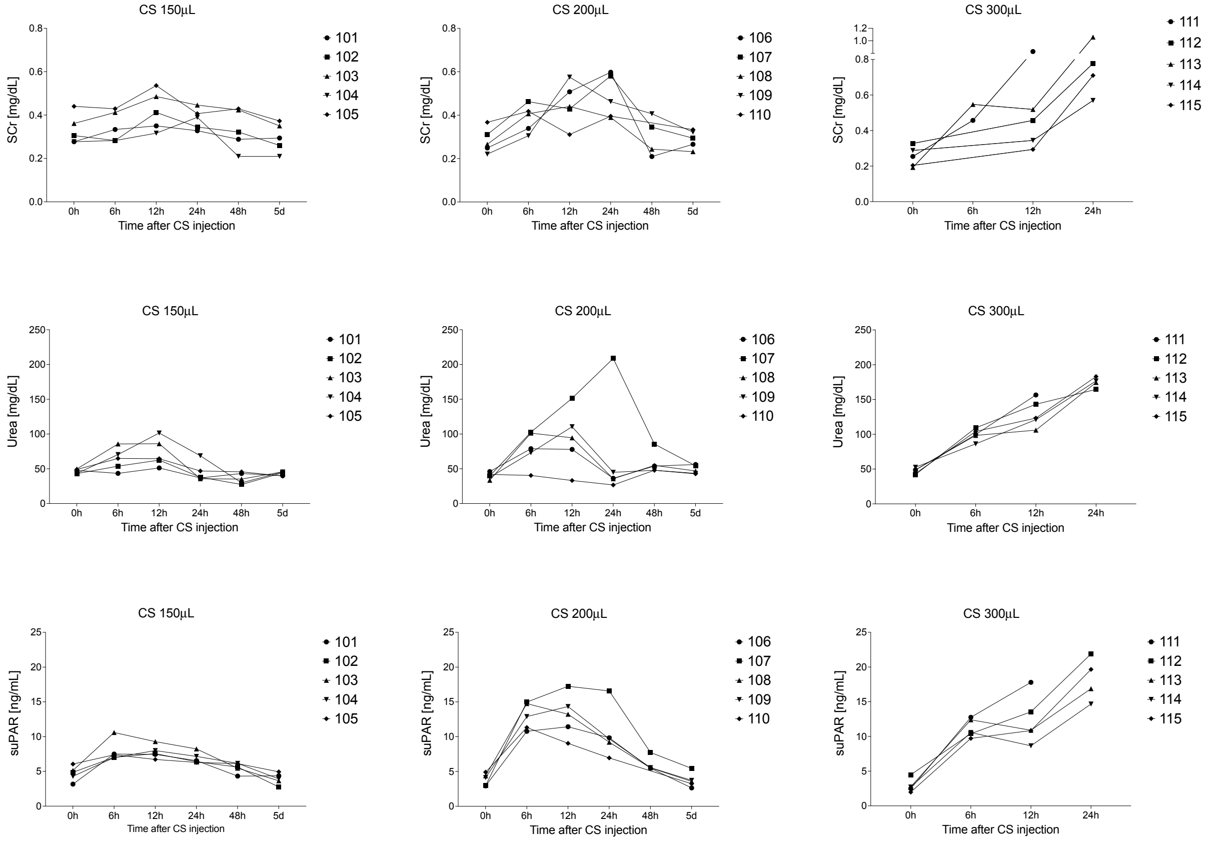

Supplemental Figure 10: Increasing volumes of intraperitoneal cecal slurry are associated with increased blood levels of soluble urokinase plasminogen activator receptor, progressive impairment of kidney function and poor survival. Dose-dependent course of serum creatinine (SCr), urea and soluble urokinase plasminogen activator receptor (suPAR) in C57BL/6 wild-type over time. Sepsis was induced via i.p. injection of 150 µL, 200 µL or 300 µL cecal slurry (CS, 6.31×10^4/ml colony forming units). Mice were followed-up for five consecutive days. Mice receiving 150µl and 200µl CS survived over 5 days, whereas all animals receiving 300µl died within 24 hours. Blood cultures taken in a sterile condition were bacteria positive in 1 of 5, 4 of 5, and 4 of 4 (one animal died before blood collection) with 150µl, 200µl, and 300µl CS, respectively.

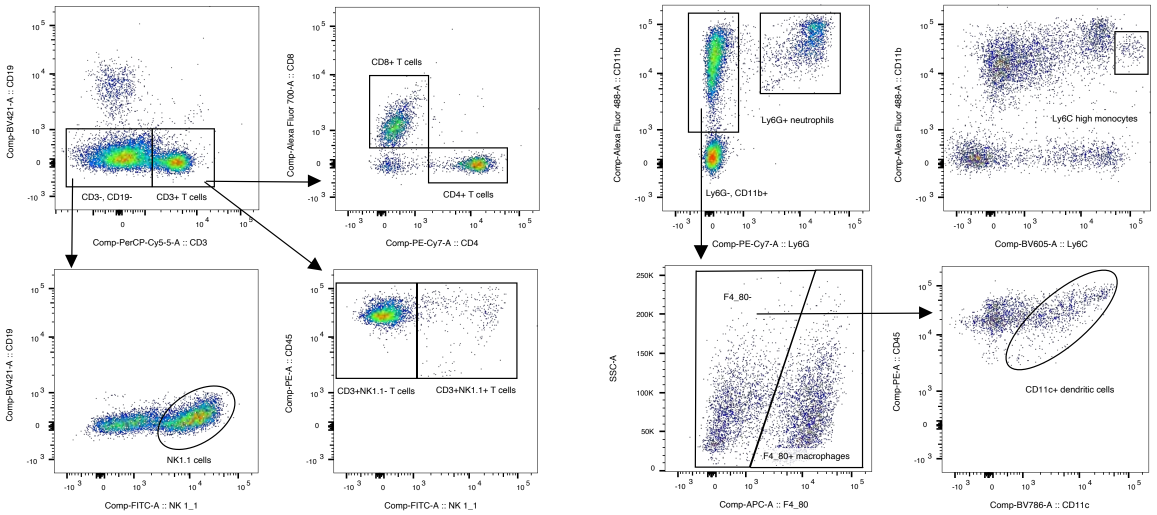

Supplemental Figure 11: Gating strategies for kidney single cell suspensions labeled with fluorophore-conjugated anti-mouse antibodies. All cell populations were gated as live single $CD45^{+}$ cells and further characterized with their respective markers. Kidney neutrophils were gated as $CD11b^{+}$ $Ly6G^{+}$ cells. Kidney monocytes were gated as $CD11b^{+}Ly6C^{high}$ cells. Kidney macrophages were gated as $Ly6G^{-}$ $CD11b^{+}$ $F4/80^{+}$. Kidney dendritic cells were gated as $Ly6G^{-}$ $CD11b^{+}$ $F4/80^{-}$ $CD11c^{+}$. Kidney T cells were gated as $CD19^{-}$, $CD3^{+}$ and then further characterized in $CD4^{+}$ and $CD8^{+}$ T cells. Natural killer (NK) cells were gated as $CD3^{-}$ $CD19^{-}$ $NK1.1^{+}$ (NK1_1). NK T cells were gated as $CD19^{-}$ $CD3^{+}$ $NK1.1^{+}$.

Supplemental tables

Supplemental Table 1: Baseline characteristics and outcomes within 7 days stratified by suPAR quartiles.

Variable	1. Quartile (n=50)	IQR (n=100)	4.Quartile (n=50)	P value
**Demographics**				
Age, years	67.9 (11.7)	65.5 (12.2)	62.8 (12.5)	0.117
Male gender, n (%)	36 (72.0)	66 (66.0)	26 (52.0)	0.096
BMI, kg/m^2	27.0 (5.9)	28.6 (7.8)	29.4 (9.4)	0.301
**Pre-existing comorbidities, n (%)**				
Chronic kidney disease (eGFR < 60ml/min/1.73 m^2)	6 (12.0)	30 (30.0)	13 (26.0)	0.052
Hypertension	31 (62.0)	75 (75.0)	39 (78.0)	0.147
Diabetes mellitus	12 (24.0)	32 (32.0)	17 (34.0)	0.499
Coronary heart disease	5 (10.0)	20 (20.0)	9 (18.0)	0.300
Congestive heart failure	3 (6.0)	10 (10.0)	8 (16.0)	0.257
Chronic obstructive pulmonary disease	6 (12.0)	17 (17.0)	4 (8.0)	0.295
SCr baseline prior sepsis, mg/dL	0.79 (0.21)	0.92 (0.35)	0.85 (0.33)	0.038
**Kidney parameters at enrollment**				
suPAR, ng/mL	4.0 (0.9)	8.2 (2.1)	19.5 (7.5)	<0.001
NGAL, ng/mL	401.5 (209.1)	670.6 (612.0)	1106.1 (915.0)	<0.001
TIMP2*IGFBP7#, $(ng/mL)^2/1000$	0.95 (1.51)	3.24 (4.97)	6.08 (10.02)	<0.001
KIM-1, pg/mL	283.7 (314.7)	333.7 (284.6)	583.9 (629.4)	0.003
SCr, mg/dL	1.35 (1.04)	1.71 (0.88)	2.28 (1.40)	<0.001
Urea, mg/dL	52.9 (31.6)	65.9 (33.0)	88.0 (46.0)	<0.001
Proteinuria, g/L	0.4 (0.4)	0.9 (1.3)	1.4 (1.5)	<0.001
Albuminuria, mg/L	64.3 (106.1)	240.5 (610.8)	501.9 (795.9)	<0.001
Urine output first 24h /L	2.5 (1.2)	2.3 (1.5)	1.9 (1.7)	0.147
**Inflammation parameters at enrollment**				
Blood leukocytes, 1/nL	12.8 (8.2)	17.8 (14.3)	17.7 (11.1)	0.050
CRP, mg/dL	194.0 (106.8)	220.8 (106.9)	217.1 (108.5)	0.339
PCT, ng/mL	16.0 (45.5)	33.2 (56.2)	48.6 (65.1)	0.016
**Primary source of sepsis, n (%)**				
Abdomen	34 (68.0)	71 (71.0)	22 (44.0)	0.004
Lung	15 (30.0)	24 (24.0)	23 (46.0)	0.023
Urinary tract	1 (2.0)	10 (10.0)	7 (14.0)	0.098
Others	5 (10.0)	6 (6.0)	3 (6.0)	0.631
**Baseline scores**				
SOFA	10.1 (3.6)	11.2 (3.5)	14.0 (3.6)	<0.001
SAPS II	58.8 (18.4)	64.0 (19.6)	71.7 (19.9)	0.004
APACHE II	27.3 (8.4)	30.5 (8.7)	33.1 (7.9)	0.003
**Outcomes**				
Cumulative fluid balance first 24h, L	1.7 (3.0)	2.9 (4.3)	5.2 (5.7)	<0.001
Septic shock, n (%)	35 (70.0)	80 (80.0)	49 (98.0)	<0.001
Invasive ventilation, n (%)	42 (84.0)	85 (85.0)	45 (90.0)	0.633
Vasopressor support, n (%)	43 (86.0)	96 (96.0)	49 (98.0)	0.020
Length of ICU stay, days	19.9 (21.5)	20.7 (22.9)	23.6 (26.6)	0.698

Length of hospital stay, days	46.7 (36.6)	43.7 (39.1)	39.5 (37.9)	0.640
Mortality 7 days, n (%)	2 (4.0)	5 (5.0)	12 (24.0)	<0.001

APACHE II = Acute Physiology and Chronic Health Evaluation II, BMI = body mass index, CRP = C-reactive protein, PCT = procalcitonin, RRT = renal replacement therapy, SAPS II = Simplified Acute Physiology Score II, SCr = serum creatinine, suPAR = soluble urokinase plasminogen activator receptor, SOFA = Sequential Organ Failure Assessment. Data are reported as mean (SD) unless otherwise indicated. # Values missing in 3 patients due to insufficient urine output. For pairwise comparisons, the two-tailed Student's t-test (continuous variables) or the chi-square test (categorial variables) were used.

Supplemental Table 2: Determinants of incident renal replacement therapy or death and major adverse kidney events within 7 days of sepsis diagnosis exclusively in patients with moderate or severe AKI at the time of enrollment (n=116)

Parameters for Adjustment	"RRT or Death" within 7d			"MAKE$_7$" within 7d		
	Odds ratio	95% CI	P value	Odds ratio	95% CI	P value
**suPAR >12.7 ng/mL**	7.11	2.49-20.33	<0.001	3.71	1.1-12.62	0.036
**SCr at enrollment**	1.52	1.00-2.32	0.048	1.15	0.75-1.76	0.529
**Age**	1.05	1.00-1-10	0.026	1.04	1.01-1.08	0.027
**Male gender**	1.01	0.37-2-74	0.982	0.88	0.33-2.38	0.803
**CKD**	0.83	0.29-2-31	0.727	1.84	0.61-5.54	0.276
**Septic shock**	2.61	0.36-18.67	0.340	3.77	0.88-16.19	0.075

Binary logistic regression analysis for suPAR levels at baseline adjusted for serum creatinine at baseline (SCr), age, gender, chronic kidney disease (CKD) and septic shock. MAKE$_7$ = major adverse kidney events within 7 days of sepsis diagnosis, RRT = renal replacement therapy. Multivariable binary logistic regression models were used to adjust for potential confounders.

Supplemental Table 3: Receiver-operating characteristic (ROC) analysis for the prediction of "RRT" within 7 days of sepsis diagnosis

Biomarker at enrollment	AUC-ROC for "RRT" within 7d		
	AUC (95% CI)	P value (AUC)	deLong's (p) toward SCr + suPAR
**SCr + suPAR**	0.86 (0.79-0.93)	<0.001	-
**SCr+NGAL**	0.85 (0.79-0.91)	<0.001	0.814
**SCr+TIMP2*IGFBP7#**	0.82 (0.75-0.90)	<0.001	0.271
**SCr+KIM-1**	0.79 (0.71-0.87)	<0.001	0.038
**SCr+Albuminuria**	0.80 (0.73-0.88)	<0.001	0.038
**suPAR**	0.82 (0.74-0.90)	<0.001	0.055
**NGAL**	0.78 (0.68-0.88)	<0.001	0.177
**TIMP2*IGFBP7#**	0.75 (0.64-0.85)	<0.001	0.070
**KIM-1**	0.51 (0.40-0.62)	0.815	<0.001
**SCr**	0.78 (0.70-0.86)	<0.001	0.011
**Proteinuria**	0.68 (0.58-0.78)	0.001	0.001
**Albuminuria**	0.69 (0.60-0.79)	<0.001	0.002

AUC = Area under the ROC curve, KIM-1 = kidney injury molecule-1, NGAL = Neutrophil gelatinase-associated lipocalin, RRT = renal replacement therapy, SCr = serum creatinine, suPAR = soluble urokinase plasminogen activator receptor, TIMP2*IGFBP7 = Tissue inhibitor of metalloproteinases 2 * Insulin-like growth factor-binding protein 7. # Values missing in 3 patients due to insufficient urine output. Diagnostic performance was assessed by receiver operating characteristics analyses. deLong's test were used for comparison of areas under the receiver operating characteristics curves (AUC-ROC).

Supplemental Table 4: Receiver-operating characteristic (ROC) analysis for the prediction of "death" within 7 days of sepsis diagnosis

Biomarker at enrollment	AUC-ROC for "Death" within 7d		
	AUC (95%-CI)	p-value (AUC)	deLong's (p) toward suPAR
**suPAR**	0.78 (0.67-0.89)	<0.001	-
**SCr**	0.59 (0.47-0.71)	0.190	0.014
**NGAL**	0.68 (0.51-0.84)	0.069	0.209
**TIMP2*IGFBP7#**	0.59 (0.44-0.74)	0.197	0.024
**KIM-1**	0.45 (0.31-0.58)	0.084	0.031
**Proteinuria**	0.57 (0.42-0.72)	0.317	0.010
**Albuminuria**	0.63 (0.49-0.77)	0.067	0.043

AUC = Area under the ROC curve, KIM-1 = kidney injury molecule-1, NGAL = Neutrophil gelatinase-associated lipocalin, SCr = serum creatinine, suPAR = soluble urokinase plasminogen activator receptor, TIMP2*IGFBP7 = Tissue inhibitor of metalloproteinases 2 * Insulin-like growth factor-binding protein 7. # Values missing in 3 patients due to insufficient urine output. Diagnostic performance was assessed by receiver operating characteristics analyses. deLong's test were used for comparison of areas under the receiver operating characteristics curves (AUC-ROC).

References

1. Nusshag C et al. Cell Cycle Biomarkers and Soluble Urokinase-Type Plasminogen Activator Receptor for the Prediction of Sepsis-Induced Acute Kidney Injury Requiring Renal Replacement Therapy: A Prospective, Exploratory Study. *Crit Care Med* 2019;47(12):e999–e1007.

2. Duff S, Murray PT. Defining Early Recovery of Acute Kidney Injury. *Clin J Am Soc Nephro* 2020;CJN.13381019.

3. Gaudry S et al. Initiation Strategies for Renal-Replacement Therapy in the Intensive Care Unit.. *New England Journal of Medicine* 2016;375(2):122–133.
